# Effects of haemodynamically atrio‐ventricular optimized His bundle pacing on heart failure symptoms and exercise capacity: the His Optimized Pacing Evaluated for Heart Failure (HOPE‐HF) randomized, double‐blind, cross‐over trial

**DOI:** 10.1002/ejhf.2736

**Published:** 2023-02-23

**Authors:** Zachary I. Whinnett, Matthew J. Shun‐Shin, Mark Tanner, Paul Foley, Badri Chandrasekaran, Philip Moore, Shaumik Adhya, Norman Qureshi, Amal Muthumala, Rebecca Lane, Aldo Rinaldi, Sharad Agarwal, Francisco Leyva, Jonathan Behar, Sukh Bassi, Andre Ng, Paul Scott, Rachana Prasad, Jon Swinburn, Joseph Tomson, Amarjit Sethi, Jaymin Shah, Phang Boon Lim, Andreas Kyriacou, Dewi Thomas, Jenny Chuen, Ravi Kamdar, Prapa Kanagaratnam, Myril Mariveles, Leah Burden, Katherine March, James P. Howard, Ahran Arnold, Pugazhendhi Vijayaraman, Berthold Stegemann, Nicholas Johnson, Emanuela Falaschetti, Darrel P. Francis, John G.F. Cleland, Daniel Keene

**Affiliations:** ^1^ National Heart and Lung Institute Imperial College London London UK; ^2^ Imperial College Healthcare NHS Trust London UK; ^3^ West Sussex Hospitals NHS Trust West Sussex UK; ^4^ Great Western Hospitals NHS Foundation Trust Swindon UK; ^5^ West Hertfordshire Hospitals NHS Trust Hertfordshire UK; ^6^ Barts Health NHS Trust London UK; ^7^ Medway NHS Foundation Trust Kent UK; ^8^ Wycombe General Hospital High Wycombe UK; ^9^ North Middlesex University Hospital London UK; ^10^ Royal Brompton and Harefield NHS Trust London UK; ^11^ Guy's and St. Thomas's NHS Foundation Trust London UK; ^12^ Royal Papworth Hospital NHS Foundation Trust Cambridge UK; ^13^ University Hospitals Birmingham Birmingham UK; ^14^ Sherwood Forest Hospitals NHS Foundation Trust UK; ^15^ Department of Cardiovascular Sciences University of Leicester Leicester UK; ^16^ Kings College NHS Hospital London UK; ^17^ Kettering General Hospital Northampton UK; ^18^ Royal Berkshire NHS Foundation Trust Reading UK; ^19^ Royal Free London Foundation NHS Trust London UK; ^20^ London North West University Healthcare NHS Trust London UK; ^21^ Sheffield Teaching Hospitals NHS Foundation Trust, Sheffield UK; ^22^ Morriston Hospital Regional Cardiac Centre Wales UK; ^23^ Nottingham University Hospitals NHS Trust Nottingham UK; ^24^ Croydon NHS University Hospital London UK; ^25^ Geisinger Commonwealth School of Medicine Geisinger Heart Institute Scranton PA USA; ^26^ Imperial College Trials Unit Imperial College London London UK; ^27^ School of Health and Wellbeing University of Glasgow Glasgow UK

**Keywords:** His bundle pacing, Atrio‐ventricular optimization, Randomized controlled trial, PR prolongation, Heart failure

## Abstract

**Aims:**

Excessive prolongation of PR interval impairs coupling of atrio‐ventricular (AV) contraction, which reduces left ventricular pre‐load and stroke volume, and worsens symptoms. His bundle pacing allows AV delay shortening while maintaining normal ventricular activation. HOPE‐HF evaluated whether AV optimized His pacing is preferable to no‐pacing, in a double‐blind cross‐over fashion, in patients with heart failure, left ventricular ejection fraction (LVEF) ≤40%, PR interval ≥200 ms and either QRS ≤140 ms or right bundle branch block.

**Methods and results:**

Patients had atrial and His bundle leads implanted (and an implantable cardioverter‐defibrillator lead if clinically indicated) and were randomized to 6 months of pacing and 6 months of no‐pacing utilizing a cross‐over design. The primary outcome was peak oxygen uptake during symptom‐limited exercise. Quality of life, LVEF and patients' holistic symptomatic preference between arms were secondary outcomes. Overall, 167 patients were randomized: 90% men, 69 ± 10 years, QRS duration 124 ± 26 ms, PR interval 249 ± 59 ms, LVEF 33 ± 9%. Neither peak oxygen uptake (+0.25 ml/kg/min, 95% confidence interval [CI] −0.23 to +0.73, *p* = 0.3) nor LVEF (+0.5%, 95% CI −0.7 to 1.6, *p* = 0.4) changed with pacing but Minnesota Living with Heart Failure quality of life improved significantly (−3.7, 95% CI −7.1 to −0.3, *p* = 0.03). Seventy‐six percent of patients preferred His bundle pacing‐on and 24% pacing‐off (*p* < 0.0001).

**Conclusion:**

His bundle pacing did not increase peak oxygen uptake but, under double‐blind conditions, significantly improved quality of life and was symptomatically preferred by the clear majority of patients. Ventricular pacing delivered via the His bundle did not adversely impact ventricular function during the 6 months.

## Graphical abstract

PR prolongation impairs atrio‐ventricular (AV) coupling: reducing left ventricular (LV) pre‐load and stroke volume, and worsening symptoms. His bundle pacing allows AV delay shortening while maintaining normal ventricular activation. HOPE‐HF evaluated whether AV optimized His pacing is preferable to no‐pacing, in randomized double‐blind cross‐over fashion. BP, blood pressure; LVEF, left ventricular ejection fraction; MLWHF, Minnesota Living With Heart Failure; QOL, quality of life; QRSd, QRS duration; RBBB, right bundle branch block; SBP, systolic blood pressure.

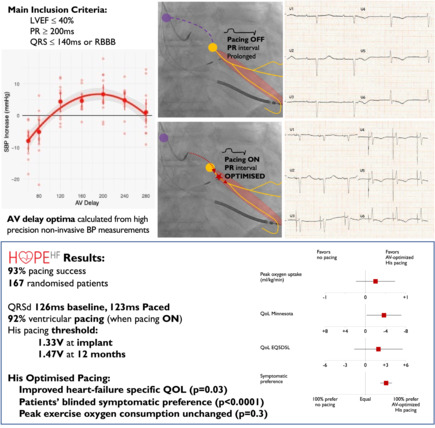

## Introduction

Excessive prolongation of atrio‐ventricular (AV) conduction causes the atria to relax before the ventricles begin to contract, leading to diastolic mitral regurgitation, a fall in left ventricular (LV) pre‐load and a lower stroke volume that, independent of left ventricular ejection fraction (LVEF), is associated with worse symptoms and a higher mortality.^1^, ^2^, ^3^ Post‐hoc analyses of two large randomized controlled trials of cardiac resynchronization therapy (CRT)^1^, ^4^ found that patients with a longer PR interval at baseline had the greatest benefit from CRT. This was not however seen in a post‐hoc analysis of the REVERSE study of CRT in those with only mild heart failure. The potential beneficial mechanism might arise as shortening pathologically prolonged PR intervals, can increase LV filling and stroke volume.^5^, ^6^ Whilst AV delay can be shortened by pacing either or both ventricles, the resulting ventricular activation pattern is not physiological, which may adversely affect ventricular function unless native ventricular activation is even worse, i.e. left bundle branch block (LBBB) or a very wide QRS.^7^

His bundle pacing can shorten AV conduction whilst maintaining normal LV activation via the native conduction system. For patients without LBBB, using His bundle pacing to shorten AV delay has improved haemodynamics acutely without inducing ventricular dyssynchrony.^8^ To investigate longer‐term clinical benefits we designed a multicentre, randomized, double‐blind, cross‐over trial (HOPE‐HF) to test the hypothesis that AV optimized His bundle pacing for patients with heart failure and an LVEF ≤40% who had a long intrinsic PR interval and narrow QRS or right bundle branch block (RBBB), would improve exercise capacity, symptoms and quality of life.

## Methods

The HOPE‐HF trial (ClinicalTrials.gov identifier: NCT02671903) was an investigator‐initiated, multicentre, randomized, blinded, cross‐over trial with AV optimized His bundle pacing programmed on compared to off. It was funded by the British Heart Foundation with excess device costs met by Medtronic. The methods have previously been described in detail^9^ and are explained briefly here.

### Participants

Patients with symptomatic heart failure and LVEF ≤40%, PR interval ≥200 ms, and either narrow QRS (≤140 ms) or RBBB (of any QRS duration) were eligible. Patients with an LVEF between 36% and 40% were required to have a plasma B‐type natriuretic peptide (BNP) >250 pg/ml.

Patients with permanent or persistent atrial fibrillation (AF) were excluded as these patients have no coordinated atrial activity. Patients with paroxysmal AF (with sustained episodes lasting >24 h) were only eligible once treatment had maintained sinus rhythm for at least 6 months. Patients were recruited from 25 centres across the UK.

### Intervention

His bundle pacing was attempted in all patients. The aim was to achieve His bundle capture without prolongation of LV activation time. We accepted either selective or non‐selective capture. Our protocol specified that if it was not possible to capture the His bundle, a LV lead should be placed instead.

Patients also received a right atrial lead and a third lead which was either a right ventricular defibrillator lead (for patients with a defibrillator indication) or an LV coronary sinus lead (for those without a defibrillator indication). The purpose of the LV lead was to allow backup pacing in the ‘no pacing’ period and an alternative pacing lead if the His lead failed in the pacing period.

### Study endpoints

The primary endpoint was peak oxygen uptake on cardiopulmonary exercise testing using a smooth modified Bruce treadmill protocol.^10^ Secondary endpoints included quality of life measured using the Minnesota Living with Heart Failure Questionnaire (MLHFQ) and the EQ‐5D visual analogue scale (VAS).

At the study visit at the end of the second period, participants were asked, under double‐blind conditions, for a binary response which of the two 6‐month trial periods were preferable in terms of heart failure symptoms.

To assess safety, in light of previous studies reporting impairment of LV function with chronic ventricular pacing, LV dimensions, LVEF and plasma BNP (Abbott ARCHITECT immunoassay BNP; Abbott Laboratories) were assessed. Hospitalizations and mortality were recorded.

### Patient flow and follow‐up

After lead and generator implantation, pacing was programmed off (apart from back‐up pacing at 30 bpm that did not use the His lead). Participants then attended the core exercise laboratory at the Hammersmith Hospital (London) for baseline assessments prior to randomization. All participants underwent haemodynamic optimization of AV delay, using the His lead, with beat‐to‐beat, non‐invasive blood pressure measurements. We have previously described^11^ why large numbers of repeated alternations are needed for a precise optimization and demonstrated how this process and analysis can be automated.^11^, ^12^ In brief, each paced and sensed AV delay is compared against a fixed reference state; in this study the reference state was intrinsic conduction. We tested a range of AV delays from 40 ms to intrinsic conduction in 40 ms intervals, both during atrial sensing and atrial pacing and thereby a curve of haemodynamic response is thereby constructed for both atrial pacing and atrial sensing. From this curve, the peak is derived. This reveals the pacing configuration that causes the greatest generation of systolic blood pressure. Participants were then randomized 1:1 to pacing programmed on (using the AV delay optimum derived as above) or off. At the end of the first 6‐month period, participants returned to the central trial hub for reassessment, including reassessment of the optimal pacing configuration. Participants then crossed over to the opposite treatment. During the His pacing on period devices had rate adaptive AV delay modification disabled and patients were programmed to DDD programming as opposed to rate adaptive programming.

### Sample size

The smoothed modified Bruce protocol in patients with heart failure has a test–retest variability of 2.4 ml/kg/min.^10^ CRT for LBBB increases peak oxygen uptake by ∼1.5 ml/kg/min.^13^, ^14^ Acute haemodynamic data suggest that the effect of His bundle pacing in patients with a long PR but narrow QRS is approximately 60% of this effect.^8^ Using a paired *t*‐test, in order to detect a difference of 0.7 with standard deviation (SD) of 2.4 with 90% power and two‐sided alpha of 0.05, suggested that 126 evaluable patients would be needed. Allowing for a combined mortality and dropout rate of 21%, the total sample size is 160 patients.

Following a review on data completeness in November 2018, there was evidence to suggest that the mortality/dropout rate was higher than the 21% originally stated. To ensure the study was adequately powered an additional seven patients were randomized into the study (based on a minimum of 188 patients being enrolled, with a minimum of 172 proceeding to implant).

### Randomization and allocation concealment

Both the patients and the staff conducting endpoint assessments were blinded to whether the His pacing was programmed on or off. The special precautions to prevent blinded staff from becoming unblinded have been previously described.^9^

### Analysis plan

The full Statistical Analysis Plan is included in online supplementary *Appendix* *S1*. Trial oversight and monitoring was delivered by the Imperial College Clinical Trials Unit. Study data were recorded in Inform, version 4.6.

All analysis is on the intention‐to‐treat principle. For continuous variables we used a generalized linear mixed model. All available data were included under the assumption of missing at random. The model included treatment, period and sequence effect as fixed effects and a random effect for inter‐subject variability. Differences in secondary outcomes were analysed using the same methodology. Normality was checked and appropriate transformation or non‐parametric methods used if needed. Sensitivity analysis was used to investigate the potential effect of missing data on the results of the primary analysis. Based on participant arm and treatment period, data were imputed based on the highest and lowest values provided in that period for that particular treatment arm and the analysis was re‐run with both the high and low imputed values. Analyses were performed using PROC MIXED with SAS version 9.4.

## Results

### Patient flow

Of 198 patients enrolled, two died, seven withdrew consent and seven were withdrawn by the investigators prior to device implantation. Five patients were withdrawn by the operator during the device implantation procedure: two because of venous stenoses preventing adequate vascular access, two because His pacing could not be achieved with acceptable parameters, and one because of peri‐procedural haemodynamic instability.

Of the 177 patients who were implanted (166 [94%] received a CRT‐D device), 176 received a His bundle lead and one patient received an LV lead. Of these 177 patients, 10 were withdrawn in the 2‐month run‐in period (pacing programmed off) before randomization: three died (one pulseless electrical activity cardiac arrest, one unrelated sepsis, and one related to previously undiagnosed cancer), two withdrew consent, one was lost to follow‐up, one withdrew due to a high His capture threshold and did not consent to lead revision and three were withdrawn by local PIs (worsening comorbidity preventing walking, development of permanent AF and pocket infection requiring system explant without subsequent reimplantation).

**Figure 1:**
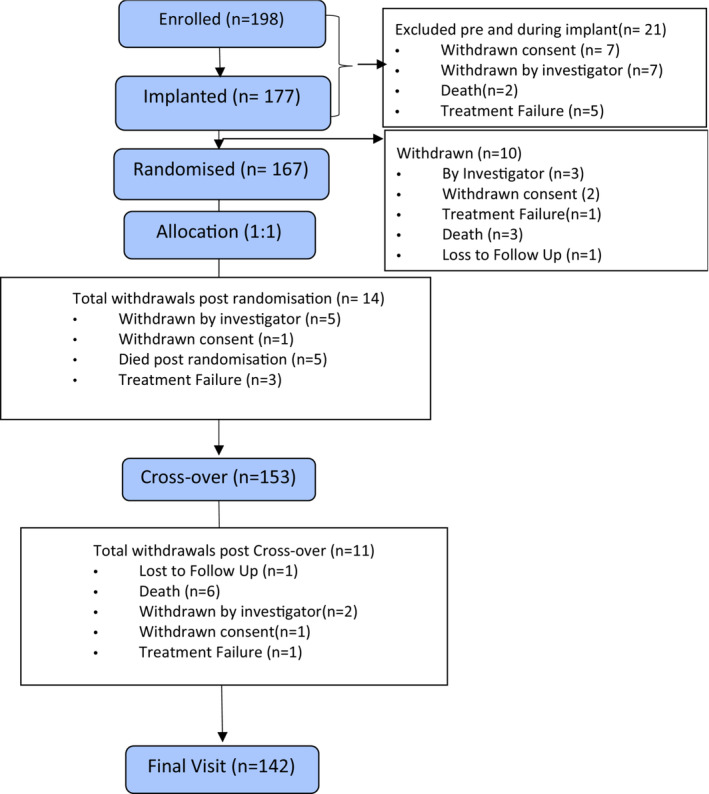
Consort flow diagram. The study enrolled 198 patients, 178 were implanted with a device in the trial and 167 underwent randomization.

After the run‐in period, 167 participants were randomized (*Table* *1*) with a mean LVEF of 33%, mean PR interval of 249 ms and mean QRS duration 124 ms; 142 completed the trial protocol (*Figure* *1*). The disposition (including mortality aetiology where relevant) of the remaining 25 is given in the online supplementary *Appendix* *S1*.

**Table 1:** Clinical characteristics of the intention‐to‐treat population at randomization

Characteristic	All patients (*n* = 167)	His pacing first (*n* = 83)	No pacing first (*n* = 84)
Male sex	151 (90)	74 (89)	77 (92)
Age (years)	69 ± 9.7	70 ± 9.0	68 ± 10.2
Ethnicity			
Asian	17 (10)	10 (12)	7 (8)
Black	8 (5)	3 (4)	5 (6)
Mixed	2 (1)	0	2 (2)
Other	3 (2)	2 (2)	1 (1)
White	137 (82)	68 (82)	69 (82)
Weight (kg)	87 ± 17.3	85 ± 16.8	89 ± 17.6
BMI (kg/m^2^)	29 ± 5.3	28 ± 5.0	30 ± 5.5
Peak oxygen uptake (ml/kg/min)	14.2 ± 4.18	13.9 ± 4.17	14.5 ± 4.19
QRS duration (ms)	124 ± 26	126 ± 28.4	121 ± 24.0
PR interval (ms)	249 ± 59.2	243 ± 51.2	254 ± 66.0
Right bundle branch block	33 (20)	20 (24)	13 (15)
LV ejection fraction (%)	33.2 ± 9.0	32.6 ± 9.7	33.8 ± 8.3
LV end‐diastolic dimension (mm)	60.3 ± 8.4	60.3 ± 7.7	60.3 ± 9.1
LV end‐systolic dimension (mm)	49.8 ± 9.9	50.0 ± 9.3	49.5 ± 10.5
BNP (ng/L)	309 [145–743]	303 [151–839]	315 [129–688]
EQ‐5D VAS	68 [50–80]	65 [50–80]	70 [50–80]
MLHF score	33 [15–81]	31 [12–50]	34 [17–53]
NYHA class			
I	2 (1)	2 (2)	0
II	134 (80)	66 (80)	68 (81)
III	30 (18)	15 (18)	15 (18)
IV	1 (1)	0	1 (1)
Ischaemic aetiology of HF	110 (66)	60 (72)	50 (60)
Diabetes	64 (38)	34 (41)	30 (36)
Hypertension	76 (46)	38 (46)	38 (45)
Smoking	37 (22)	19 (23)	18(21)
History of atrial fibrillation	31 (19)	18 (22)	13 (16)
ACE inhibitors/A2RAs	98 (59)	46 (55)	52 (62)
Aldosterone antagonists	90 (54)	38 (46)	52 (62)
Beta‐blockers	113 (68)	53 (64)	60 (71)
Diuretics	80 (48)	39 (47)	41 (49)
Sacubitril/valsartan	15 (9)	7 (8)	8 (10)

Values are given as *n* (%), mean ± standard deviation, or median [interquartile range].

A2RA, angiotensin II receptor antagonists; ACE, angiotensin‐converting enzyme; BMI, body mass index; BNP, B‐type natriuretic peptide; HF, heart failure; LV, left ventricular; MLHFQ, Minnesota Living with Heart Failure Questionnaire; NYHA, New York Heart Association; VAS, visual analogue scale.

### Pacing data

Implant times were 118.9 (SD 50.4) min. No patients had to withdraw from a no‐pacing period because of a failure of AV conduction.

During the His pacing periods, the mean percentage of pacing received at the His bundle was 92% (± 18) with 75.7% of patients receiving >95%. During the His pacing periods patients received atrial pacing 26% (SD ± 32) of the time. During pacing‐off periods the proportion of ventricular (RV) pacing was 13% (SD ± 28) and atrial pacing was 16% (SD ±28). During pacing‐on periods, mean heart rate was 70 ± 9 bpm and minimum atrial rate was 53 bpm. During pacing‐off periods, these values were 70 ± 10 bpm and 47 bpm, respectively.

The mean optimal His‐paced AV delay was 195 ms (median 200, interquartile range [IQR] 170–210) and the mean optimal sensed AV delay 131 ms (median 140, IQR 100–170). Optimization protocols were performed with both atrial sensing (69 ± 10 bpm) and atrial pacing at 10 bpm above the intrinsic heart rhythm (80 ± 9 bpm). The mean intrinsic (126 ms [± 28]) and His‐paced QRS duration (123 ms [± 23]) were similar. For those with RBBB at baseline 166 ms (± 20) this shortened to 151 ms (± 29) with His bundle pacing. The His pacing thresholds at 1 ms pulse width were similar at the randomization and final follow‐up visits (1.33 ± 1.07 V and 1.47 ± 1.21 V).

Of the 179 participants in whom the His pacing procedure was attempted, His bundle pacing could not be achieved in three, one of whom received an LV lead via the coronary sinus (as described above). Ten (5.6%) others experienced a significant rise in threshold or lead displacement during the study (six pre‐randomization, three between randomization and cross‐over and one after cross‐over). Six of these patients agreed to undergo lead revision and continue with the study; the other four patients declined reintervention and exited the study. Therefore, of patients who had attempted His bundle lead implantation, this was successful at the first procedure for 93% (166/179).

### Endpoints

His bundle pacing did not increase peak exercise oxygen consumption (+0.25 ml/kg/min, 95% confidence interval [CI] −0.23 to +0.73, *p* = 0.3) but MLHFQ score improved significantly (−3.7, 95% CI −7.1 to −0.3, *p* = 0.03) although a generic quality of life score (EQ‐5D VAS) did not show a statistically significant improvement (+1.9, 95% CI −1.6 to +5.5, *p* = 0.28). Further exercise endpoints as well as analysis at different time points are detailed in the online supplementary *Appendix* *S1*.

### Safety endpoints

Left ventricular dimensions, LVEF, plasma BNP nor incidence of ventricular arrhythmias did not change significantly with His bundle pacing.

### Patient preference

Of 167 randomized participants, 148 indicated a preference for one of the two 6‐month trial periods. For 112 patients (76%) the preference was for the His bundle pacing period and for 36 (24%) it was for the no‐pacing period (*p* < 0.0001).

**Figure 2:**
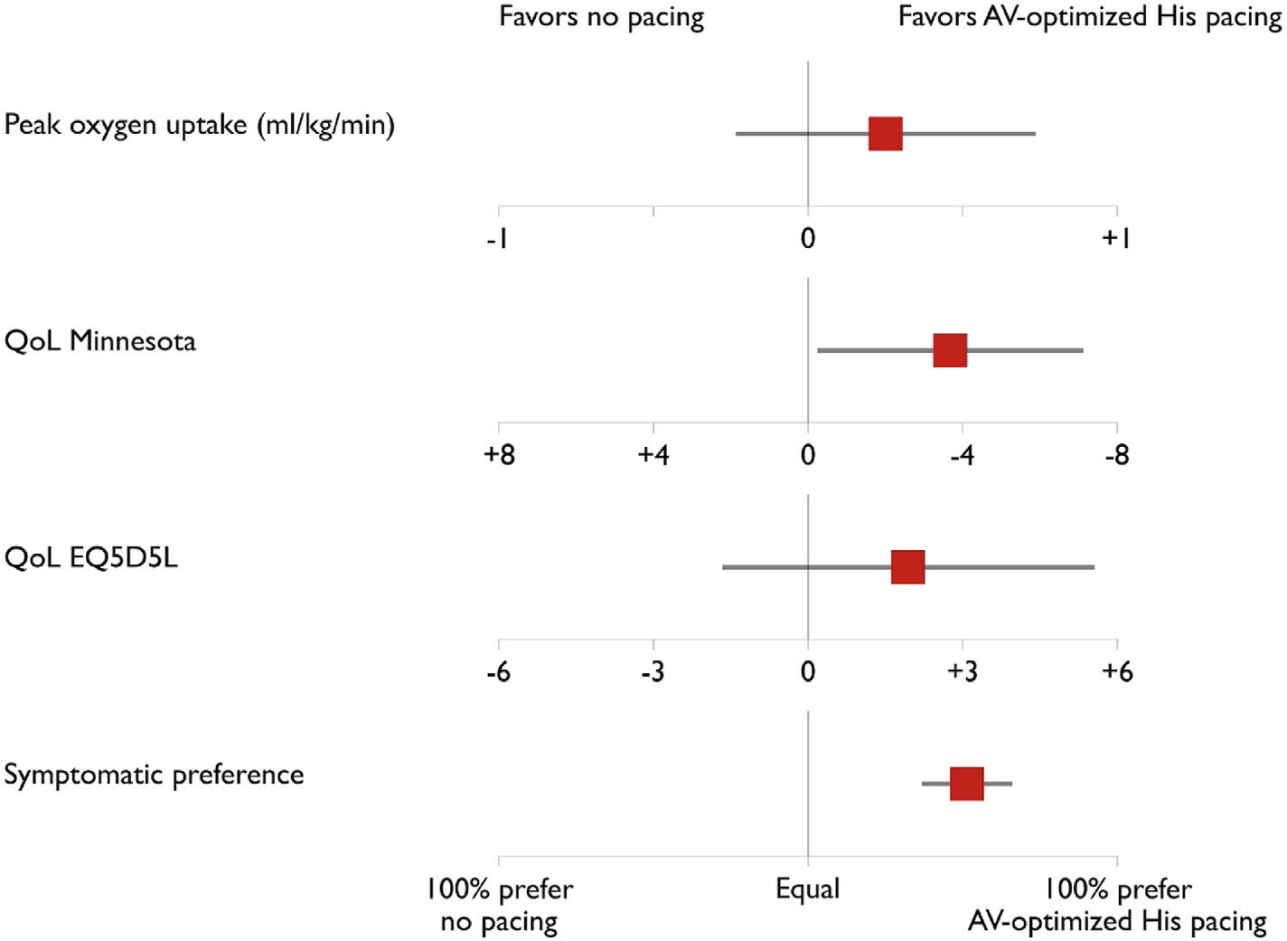
Efficacy endpoints. AV, atrio‐ventricular; QoL, quality of life.

**Figure 3:**
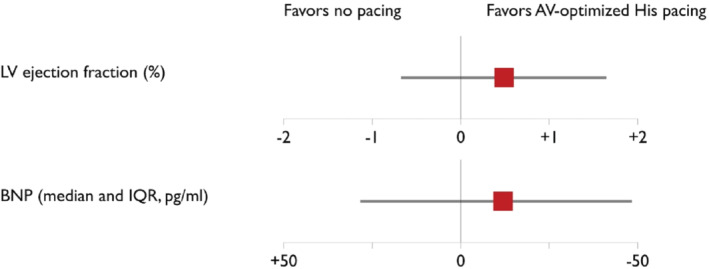
Safety endpoints. AV, atrio‐ventricular; BNP, B‐type natriuretic peptide; IQR, interquartile range; LV, left ventricular.

The mixed‐model treatment effects and endpoint results are summarized in *Table* *2* and *Figures* *2* and *3*. Additional subgroup analyses including RBBB versus non‐RBBB, narrow QRS (<120 ms) versus non narrow QRS, New York Heart Association (NYHA) class II versus NYHA class III + IV and treatment effect on NYHA class are provided in online supplementary *Appendix* *S1*.

**Table 2:** Endpoints

Characteristic	*N*	No pacing	Pacing	Δ	*p*‐value
Peak oxygen uptake (ml/kg/min)	167	13.7 (12.8, 14.6)	14.0 (13.0, 15.0)	+0.25 (−0.23, 0.73)	0.30
MLHFQ score	151	34.6 (29.8, 39.3)	30.9 (25.1, 36.7)	−3.7 (−7.1, −0.3)	0.03
EQ‐5D5L VAS	151	64.3 (60.4, 68.3)	66.3 (61.0, 71.6)	+1.9 (−1.6, 5.5)	0.28
LVEF (%)	167	33.0 (31.0, 34.9)	33.4 (31.2, 35.7)	+0.5 (−0.7, 1.6)	0.40
LVEDD (mm)	167	60.0 (58.1, 61.9)	60.1 (58.0, 62.2)	+0.1 (−0.9, 1.1)	0.83
LVESD (mm)	167	49.9 (47.8, 51.9)	50.6 (48.2, 52.9)	+0.7 (−0.4, 1.78)	0.23
BNP^a^	167	335 (257, 436)	323 (242, 431)	−12 (−48, 28)	0.54

BNP, B‐type natriuretic peptide; MLHFQ, Minnesota Living with Heart Failure Questionnaire; LVEDD, left ventricular end‐diastolic dimension; LVEF, left ventricular ejection fraction; LVESD, left ventricular end‐systolic dimension; VAS, visual analogue scale.

The Δ column shows the estimated treatment effect on each variable with its 95% confidence interval.

^a^ BNP was log‐transformed before analysis in the mixed‐model and then back transformed for presentation.

### Adverse events

Six participants asked to cross‐over before a planned 6‐month assessment. In each case, this was a transition from no pacing to His bundle pacing. During the pacing period, there were 19 heart failure admissions: 12 hospitalizations in 10 patients receiving pacing first and seven hospitalizations in six patients receiving pacing second. During the period without pacing, there were 19 heart failure admissions: four in four patients with pacing off first and 15 in 12 patients receiving pacing off second. During the 12‐month randomized period, 11 deaths occurred: six with pacing on and five with pacing off.

## Discussion

HOPE‐HF is the first substantial trial targeting isolated PR prolongation in patients with heart failure.^15^, ^16^ The results of HOPE‐HF indicate that AV optimized His bundle pacing for patients with heart failure and a reduced LVEF who have a long PR interval does not improve exercise capacity. However, it does seem to be symptomatically preferred by patients and improve heart failure specific quality of life. Furthermore, ventricular pacing delivered using His bundle pacing did not appear to adversely affect ventricular function within the 6‐month pacing period when delivered to patients with heart failure and a narrow QRS duration.

### Isolated PR prolongation as a therapeutic target

For patients with heart failure, PR prolongation is associated with a worse outcome, possibly because it leads to impaired coupling of the timing of atrial and ventricular contraction with adverse haemodynamic consequences.^1^, ^3^, ^17^ Right ventricular or biventricular pacing can shorten PR but, in the absence of pre‐existing intra‐ventricular conduction delay, may cause or exacerbate ventricular dyssynchrony. Biventricular pacing is therefore only beneficial when native ventricular activation is grossly disturbed, such as with LBBB^7^ or when there is a bradycardia pacing indication and the alternative is right ventricular pacing. His bundle pacing permits abnormally long PR intervals to be normalized without causing ventricular dyssynchrony.

By a 3:1 majority, under double‐blind conditions, patients preferred pacing‐on rather than pacing‐off. This preference testing can be sensitive because it asks each patient to make a head‐to‐head comparison of heart failure symptoms between the two periods and report their personally prioritized judgement of which period was better. It should be remembered that while this was prospectively collected under double‐blind conditions it was not the heart failure symptom feature listed on ClinicalTrials.org and therefore should be interpreted with caution. The 3:1 ratio seen here is much smaller than the 5.7:1 ratio seen in the pioneering blinded cross‐over MUSTIC trial of biventricular pacing in LBBB.^18^, ^19^

The heart failure‐specific quality of life assessment (MLHFQ) was also statistically significantly better with pacing‐on than with pacing‐off, although the size of this improvement (3.7 units) is relatively small, it is within the spectrum of sizes of improvements seen in the five blinded trials of biventricular pacing in heart failure with broad QRS (the early trials of these paved the way for further larger trials): REVERSE^20^ 2 units, MIRACLE^21^ 9 units, MIRACLE ICD^22^ 6 units, MIRACLE ICD II^14^ 2.6 units and MUSTIC^18^ ∼14 units. The non‐heart failure‐specific tool (EQ‐5D) did not show a statistically significant difference.

We speculate that the symptomatic effect size of this intervention (PR interval optimization) is smaller than that of conventional biventricular pacing (PR interval optimization + QRS narrowing) in eligible patients because only one electrical target is being modified rather than two.

Moreover, the patients' dichotomous preferences and quality of life were judged over the entirety of their periods, whereas peak oxygen uptake is evaluated over a few seconds level of activity that patients do not attain spontaneously. This may explain why dichotomous preference and heart failure‐specific quality of life was sensitive to the effect of pacing.

### Safety: left ventricular function

Pacing therapy for heart failure has been focused on patients where the ventricular activation pattern is sufficiently abnormal (LBBB) that the available pacing techniques (biventricular pacing) produce improvements in ventricular activation time relative to intrinsic conduction. The reduction in ventricular activation time produces improved coordination of ventricular contraction, leading to an increased ejection fraction.^23^, ^24^

Shortening abnormally long PR intervals without affecting QRS, as in HOPE‐HF, is not intended to affect ventricular activation sequence or ejection fraction. HOPE‐HF showed no change in LVEF: any benefits arise not from more synchronous systole, but rather from more efficient use of the limited time available in diastole. If back‐pressure in the pulmonary circulation is contributing to symptoms, it should be alleviated by optimization of the AV delay without requiring any effect on ventricular activation sequence of systolic contraction.

DAVID and EchoCRT showed that ventricular pacing can have adverse effects on morbidity and mortality when it is delivered using right ventricular or biventricular pacing to patients with ventricular impairment and narrow intrinsic QRS duration.^15^, ^16^ Several studies^25^, ^26^ suggest that harm builds progressively over time,^16^, ^27^ an exact mirror image of the gradual benefit over time in patient groups with more seriously abnormal ventricular activation.^4^, ^23^, ^24^, ^28^, ^29^

Despite the high percentages of ventricular pacing (92%) in HOPE‐HF, although not significant, the changes in LVEF and BNP were in favour of His bundle pacing, suggesting that the ventricle can be paced at the His bundle without worsening ventricular function, in patients with normal QRS morphology and pre‐existing LV impairment.

His bundle lead implant safety and feasibility in HOPE‐HF were similar to those observed with LV leads^30^ and on par with large‐scale registry experience of His bundle pacing.^31^ Thresholds remained stable over the duration of the 14 months of participation.

Although the non‐significant trend in LVEF was numerically positive, we cannot be certain that this trend would continue favourably and become statistically significant over longer periods or in a larger study. Only a larger or longer trial could answer this question.

### Choice of endpoints versus choice of study design

It has unfortunately become common practice to treat symptoms (reported by the patient, i.e. ‘subjective’) as a less reliable guide of therapy success than measurements (reported by someone else, i.e. ‘objective’). We have previously studied this across 2074 patients in studies of CRT for narrow QRS.^32^ Remarkably it was not the choice of endpoint, but rather the choice of the study design that was the key. For studies without randomization most endpoints, be they subjective or objective, showed false positive improvements. The randomized studies consistently showed less bias. Remarkably the randomized blinded trials consistently showed no bias regardless of the nature of the endpoint, subjective or objective. This is why our study used both randomization and blinding.

### Personalized atrio‐ventricular delay programming

HOPE‐HF found that the mean haemodynamically optimal AV delay was 200 ms when the atrium was paced and 130 ms when the intrinsic atrial signal was sensed. These are longer than a nominal default frequently now used for AV delay, but shorter than the individuals' intrinsic PR intervals. Physicians seeking to replicate the results of this study should also use a similar high‐precision haemodynamic AV optimization process.

### Study limitations

Although this is the largest randomized controlled trial of conduction system pacing to date, the CIs around the effect on ventricular function and cardiopulmonary exercise test do encompass some possibility of harm. Only a larger study could exclude this possibility. Like any randomized controlled trial, it could only randomize patients meeting the criteria pre‐specified by expert peer review of the protocol, and who agreed to be randomized. It is known that patients unwilling to consider research have worse prognosis for unknown reasons.

Treatment periods were only 6 months, which allowed a cross‐over design where each participant was their own control. This increased power as intra‐individual variation is less than the variation amongst individuals. Also, as all patients had the possibility to experience the effects of treatment, they could decide which treatment period they preferred. Longer periods would have impaired accuracy of recollection and would have reduced the proportion of participants who completed the trial.

The mean PR interval was only 249 ms, and therefore the reduction in AV delay may have been relatively modest. A cohort with even longer PR intervals might have had a greater effect on heart failure symptoms and perhaps even a statistically significant effect on exercise capacity and ejection fraction.

There was only 92% ventricular pacing. In some senses, this is a limitation if one is trying to measure a theoretical maximum impact of this intervention. However, the nature of patients in heart failure and conduction system disease is that ectopy and episodes of arrhythmia are not infrequently present. What matters clinically is the effectiveness of the strategy in a realistic cohort of patients.

Our protocol used haemodynamic AV delay optimization carried out during a hospital visit just before the pacing‐on period. There was no adjustment to the programmed AV delay during the pacing‐on period. It is possible that the haemodynamic optimum changed during this time. Previous work in heart failure patients with LBBB suggests though that there is little change over 6 months.

Participants, clinicians and the researchers contacting the participants and performing the evaluations, were all blinded to allocation arm. This minimized the risk of bias. We wanted them to focus their attention on deciding on which period had better symptoms and not to take any steps to identify which arm was active and which control. Unfortunately, this means that we do not have data on which way round the periods were; all we know is that they expressed a distinct preference for the pacing period (*p* < 0.0001).

Deaths and hospitalizations were few, but this study was never intended to detect a difference in these endpoints.

There may have been a learning curve for His bundle lead implantation in some centres. HOPE‐HF did not impose a minimum experience requirement before centres or operators could implant in the trial, but procedural success rates were nevertheless satisfactory.

## Conclusions

In patients with heart failure with reduced ejection fraction and a long PR interval who are not indicated for conventional biventricular pacing, optimization of the time interval between atrial and ventricular contraction with His bundle pacing did not increase exercise peak oxygen uptake but was clearly preferred by patients (3:1 ratio) and improved heart failure‐specific quality of life. During the 6‐month period, His bundle pacing did not induce electrical ventricular dyssynchrony or impair LV systolic function. The results of this blinded physiological trial suggest that it would be worthwhile to conduct a longer‐term trial powered for event endpoints.

## Supporting information


**Appendix S1.** Supporting Information.
