# Supplementary material for: Effects of haemodynamically atrio‐ventricular optimized His bundle pacing on heart failure symptoms and exercise capacity: the His Optimized Pacing Evaluated for Heart Failure (HOPE‐HF) randomized, double‐blind, cross‐over trial
Source: Eur J Heart Fail. 2023 Feb 23;25(2):274–83. doi: 10.1002/ejhf.2736 (PMC10946926; doi:10.1002/ejhf.2736)
Supplement: Supplementary file 1 — Appendix S1. Supporting Information. [file EJHF-25-274-s001.pdf]

Supplementary Appendix

1 Disposition of the 25 randomized patients who did not complete the trial

2 Cause of Death within Trial

3 Data on device detected ventricular arrhythmias within Trial

4 Additional Exercise Tests Parameters

5 Subgroup Analyses (all endpoints)

Baseline Right Bundle Branch Block versus no Right Bundle Branch Block

Baseline NYHA II versus NYHA III and IV

Baseline QRS <120ms versus >= 120ms

Baseline EF <30% versus >= 30%

6 Treatment effect on NYHA class

7 Individual patient data on NYHA 3 patients at baseline

8 Individual patient data on RBBB patients at baseline

9 His Pacing parameters at different trial visits

10 Per Protocol Analysis

11 Analysis at the end of first visit only

12 Analysis between first and second visits

13 Statistical Analysis Plan (SAP)

1 Disposition of the 25 randomized patients who did not complete the trial

14 patients discontinued from the study between randomisation and cross-over. (1 withdrew consent, 5 died, 5 withdrawn by PI [3 acceptance of permanent AF, 1 cardiac transplant, 1 worsening comorbidity and involvement of palliative care] and 3 treatment failures [2 increased threshold/micro displacement and patient not agreeable for system revision and 1 developed CHB and PI decision to implant an LV lead as per guidelines]).

11 patients discontinued from the study between cross-over and end-of-study. (1 withdrew consent, 1 was lost from follow-up, 6 died, 2 withdrawn by PI [worsening comorbidity and involvement of palliative care] and 1 treatment failure [increased threshold/micro displacement and patient not agreeable for system revision]).

2 Cause of Death within Trial

11 deaths occurred

6 whilst patients were receiving His bundle pacing

(x2 MI, x2 Cardiac Arrest, 1 Pneumonia, 1 Renal Failure)

5 whilst patients were receiving No pacing

(x1 MI, x1 Cardiac Arrest, x1 Intra Cranial Haemorrhage, x1 Lung Ca, x1 Multiorgan Failure secondary to cardiac failure)

3 Data on device detected ventricular arrhythmias within Trial

VF Episodes

Table of VF episodes by treatment			
**VF episodes**	**treatment**		
**Frequency [n (%)]**	**AV His-bundle N=141**	**No pacing N=147**	**Total**
**0**	139 (98.6)	145 (98.6)	284
**1**	2 (1.4)	2 (1.4)	4
**Frequency Missing = 15**			

FVT episodes

Table of FVT episodes by treatment			
**FVT episodes**	**treatment**		
**Frequency [n (%)]**	**AV His-bundle N=142**	**No pacing N=147**	**Total**
**0**	137 (96.5)	142 (96.6)	279
**1**	2 (1.4)	2 (1.4)	4
**3**	1 (0.7)	-	1
**4**	-	1 (0.7)	1
**5**	-	1 (0.7)	1
**8**	-	1 (0.7)	1
**12**	1 (0.7)	-	1
**17**	1 (0.7)	-	1
**Frequency Missing = 14**			

4 Additional Exercise Tests Parameters
Summary Table of Changes (Unadjusted)

Variable	Δ Pacing	Δ No-pacing	Δ Effect	Lower CI	Upper CI
Peak Oxygen Uptake	-0.08	-0.25	0.1681	-0.5177	0.8539
Exercise Time	-12.78	-10.48	-2.3041	-17.2991	12.6910
Respiratory Exchange Ratio	-0.02	-0.02	-0.0005	-0.0289	0.0278

Δ Pacing/No-Pacing = Value at Treatment Period – Value taken at Randomisation
Δ Effect = Pacing – No Pacing

5 Subgroup Analyses

Baseline Right Bundle Branch Block versus no Right Bundle Branch Block

				Mixed Model Effect			
**Endpoint**	**N**	Term	Value	Value	LCI	UCI	p-value
MVO2	164	Treatment	AV Pacing	0.30	(-0.22,	0.82)	0.26
		Subgroup	RBBB	-0.61	(-2.15,	0.94)	0.44
		Interaction	RBBB*Pacing	-0.47	(-1.55,	0.62)	0.4

Baseline NYHA (I and) II versus NYHA III and IV

				Mixed Model Effect			
**Endpoint**	**N**	Term	Value	Value	LCI	UCI	p-value
MVO2	167	Treatment	AV Pacing	0.23	(-0.33,	0.79)	0.42
		Subgroup	NYHA III/IV	-2.18	(-3.43,	-0.92)	0.0007
		Interaction	NYHA III/IV*Pacing	0.06	(-0.82,	0.93)	0.9

Baseline QRS <120ms versus >= 120ms

				Mixed Model Effect			
**Endpoint**	**N**	Term	Value	Value	LCI	UCI	p-value
MVO2	167	Treatment	AV Pacing	0.15	(-0.50,	0.80)	0.65
		Subgroup	QRS < 120	0.59	(-0.62,	1.80)	0.34
		Interaction	QRS <120*Pacing	0.19	(-0.64,	1.01)	0.66

Baseline EF <30% versus >= 30%

				Mixed Model Effect			
**Endpoint**	**N**	Term	Value	Value	LCI	UCI	p-value
MVO2	167	Treatment	AV Pacing	-0.06	(-0.83,	0.71)	0.88
		Subgroup	EJF >= 30	1.4	(0.13,	2.67)	0.031
		Interaction	EJF >= 30*Pacing	0.45	(-0.43,	1.34)	0.31

6 Treatment effect on NYHA class

Table of treatment by NYHA CLASS				
**treatment**	**NYHA CLASS**			
**Frequency [n (%)]**	**1**	**2**	**3**	**4**
**AV optimised direct His-bundle [n=146]**	2 (1.4)	112 (76.7)	30 (20.5)	2 (1.4)
**No pacing [n=151]**	3 (2.0)	110 (72.8)	34 (22.5)	4 (2.7)
**Total [n=297]**	5 (1.7)	222 (74.7)	64 (21.5)	6 (2.0)
**Frequency Missing = 38**				

Table of treatment by NYHA GROUP		
**treatment**	**NYHA GROUP**	
**Frequency [n (%)]**	**1/2**	**3/4**
**AV optimised direct His-bundle [n=146]**	114 (78.1)	32 (21.9)
**No pacing [n=151]**	113 (74.8)	38 (25.2)
**Total [n=297]**	227 (76.4)	70 (23.6)
**Frequency Missing = 38**		

Statistic (NYHA GROUP)	DF	Value	Prob
**Chi-Square**	1	0.4346	0.5097

7 Individual patient data on NYHA 3 patients at baseline

		Arm		
**Variable**	**Statistics**	**1 - (AV Opt. Dir. / No Pacing)**	**2 - (No Pacing / AV Opt. Dir.)**	**All Subjects**
Age (y)	n	30	24	54
	Mean	71.8	67.6	69.9
	SD	9.04	11.29	10.22
	Median	73.0	68.0	71.5
	Q1	65.0	58.5	62.0
	Q3	80.0	76.5	79.0
Ethnicity (%)	Asian	1 (1.2)	1 (1.2)	2 (1.2)
	Black	1 (1.2)	0	1 (0.6)
	Mixed	0	2 (2.4)	2 (1.2)
	White	28 (33.7)	21 (25.0)	49 (29.3)
Gender (%)	Female	4 (4.8)	4 (4.8)	8 (4.8)
	Male	26 (31.3)	20 (23.8)	46 (27.5)
Height (cm)	n	30	24	54
	Mean	172.6	172.8	172.7
	SD	9.15	9.26	9.11
	Median	173.5	172.0	173.0
	Q1	166.0	167.5	166.0
	Q3	179.0	180.5	180.0
MVO2 (mls/kg/min)	n	30	24	54
	Mean	11.98	13.31	12.57
	SD	3.448	3.230	3.388
	Median	11.70	11.90	11.85
Weight (kg)	n	30	24	54
	Mean	84.79	89.93	87.08
	SD	15.082	17.020	16.026
	Median	83.10	85.80	84.00
	Q1	77.50	77.05	77.50
	Q3	91.40	102.60	100.90
BMI (kg/m2)	n	30	24	54
	Mean	28.445	30.003	29.138
	SD	4.6593	4.4841	4.6060
	Median	26.935	29.690	27.675
	Q1	25.090	27.010	26.080

		Arm		
Variable	Statistics	1 - (AV Opt. Dir. / No Pacing)	2 - (No Pacing / AV Opt. Dir.)	All Subjects
	Q3	31.460	33.500	32.590
Pulse (bpm)	n	30	24	54
	Mean	64.8	67.9	66.2
	SD	10.62	13.75	12.09
	Median	65.0	66.0	65.0
	Q1	60.0	58.5	59.0
	Q3	72.0	75.5	73.0
Diastolic BP (mmHg)	n	30	24	54
	Mean	66.8	69.2	67.9
	SD	10.66	11.48	10.99
	Median	63.7	67.2	66.3
	Q1	60.7	59.8	60.3
	Q3	72.3	77.2	76.0
Systolic BP (mmHg)	n	30	24	54
	Mean	118.6	119.9	119.1
	SD	19.09	23.65	21.04
	Median	115.3	112.2	113.3
	Q1	108.0	102.8	106.3
	Q3	128.3	134.7	130.0
Education (%)	Secondary	23 (27.7)	12 (14.3)	35 (21.0)
	University or college	5 (6.0)	6 (7.1)	11 (6.6)
	Unknown	2 (2.4)	6 (7.1)	8 (4.8)
6 Min Walk Test (m)	n	30 (36.1)	24 (28.6)	54 (32.3)
	Mean	199.70 (240.	249.79 (297.	221.96 (132.
	SD	75.896 (91.4	83.732 (99.7	82.617 (49.5
	Median	195.50 (235.	256.50 (305.	206.00 (123.

8 Individual patient data on RBBB patients at baseline

		Arm		
**Variable**	**Statistics**	**1 - (AV Opt. Dir. / No Pacing)**	**2 - (No Pacing / AV Opt. Dir.)**	**All Subjects**
Age (y)	n	19	14	33
	Mean	73.8	69.2	71.8
	SD	8.80	12.05	10.38
	Median	76.0	70.5	74.0
	Q1	65.0	61.0	65.0
	Q3	80.0	76.0	79.0
Ethnicity (%)	Asian	1 (1.2)	1 (1.2)	2 (1.2)
	Black	1 (1.2)	0	1 (0.6)
	Other Ethnic Group	1 (1.2)	0	1 (0.6)
	White	16 (19.3)	13 (15.5)	29 (17.4)
Gender (%)	Female	1 (1.2)	0	1 (0.6)
	Male	18 (21.7)	14 (16.7)	32 (19.2)
Height (cm)	n	19	14	33
	Mean	170.7	173.2	171.8
	SD	9.21	5.95	7.98
	Median	170.0	172.0	172.0
	Q1	162.0	170.0	169.0
	Q3	179.0	176.0	176.0
MVO2 (mls/kg/min)	n	19	14	33
	Mean	13.72	14.41	14.02
	SD	4.406	3.837	4.126
	Median	12.80	13.30	12.90
Weight (kg)	n	19	14	33
	Mean	81.82	87.21	84.10
	SD	16.322	14.287	15.495
	Median	80.40	85.80	83.00
	Q1	74.00	77.90	74.10
	Q3	87.90	93.30	88.50

		Arm		
Variable	Statistics	1 - (AV Opt. Dir. / No Pacing)	2 - (No Pacing / AV Opt. Dir.)	All Subjects
BMI (kg/m2)	n	19	14	33
	Mean	27.935	29.034	28.401
	SD	4.1027	4.3487	4.1779
	Median	26.820	28.360	27.820
	Q1	25.090	26.960	25.390
	Q3	29.510	30.310	30.230
Pulse (bpm)	n	19	14	33
	Mean	64.3	61.9	63.3
	SD	10.70	10.22	10.41
	Median	62.0	60.0	61.0
	Q1	55.0	59.0	58.0
	Q3	73.0	63.0	68.0
Diastolic BP (mmHg)	n	19	14	33
	Mean	65.0	69.3	66.8
	SD	11.90	9.27	10.92
	Median	62.3	70.0	63.7
	Q1	56.0	60.3	59.3
	Q3	68.0	76.7	76.0
Systolic BP (mmHg)	n	19	14	33
	Mean	119.4	120.8	120.0
	SD	15.65	23.94	19.26
	Median	114.7	116.0	114.7
	Q1	110.7	105.7	110.3
	Q3	136.0	129.0	129.0
Education (%)	Secondary	14 (16.9)	10 (11.9)	24 (14.4)
	University or college	3 (3.6)	1 (1.2)	4 (2.4)
	Unknown	2 (2.4)	3 (3.6)	5 (3.0)
smwt	n	19 (22.9)	14 (16.7)	33 (19.8)
	Mean	248.37 (299.	307.07 (365.	273.27 (163.
	SD	99.386 (119.	146.344 (174	122.982 (73.

		Arm		
**Variable**	**Statistics**	**1 - (AV Opt. Dir. / No Pacing)**	**2 - (No Pacing / AV Opt. Dir.)**	**All Subjects**
	Median	212.00 (255.	300.50 (357.	242.00 (144.

9 His Pacing parameters at different trial visits

				Summary Statistics					
**Analyte**	**Arm**	**Visit**	**Treatment**	**n**	**Mean**	**SD**	**Median**	**Min.**	**Max.**
Impedance of HIS Lead (ohms)	1	BL (Rand.)	No pacing (run-in)	82	377.915	104.2815	342.000	228.00	627.00
		M6	Pacing	73	326.301	84.2438	285.000	228.00	570.00
		M12	No-Pacing	69	324.928	87.0778	285.000	228.00	551.00
	2	BL (Rand.)	No pacing (run-in)	83	392.361	97.9404	380.000	247.00	589.00
		M6	No-Pacing	81	361.704	91.0933	323.000	228.00	551.00
		M12	Pacing	72	330.556	82.6823	304.000	209.00	532.00
Sensed His-Amplitude (mV)	1	BL (Rand.)	No pacing (run-in)	6	6.100	2.8531	6.100	1.80	9.30
		M6	Pacing	1	1.600	.	1.600	1.60	1.60
		M12	No-Pacing	0	.	.	.	.	.
	2	BL (Rand.)	No pacing (run-in)	9	5.400	2.7005	6.300	0.60	7.60
		M6	No-Pacing	7	3.964	2.3428	4.100	0.60	6.30
		M12	Pacing	3	4.433	3.5529	4.600	0.80	7.90

13 Statistical Analysis Plan (SAP)

Statistical Analysis Plan (SAP)

AV optimisation delivered with direct His bundle pacing, in patients with heart failure, long PR without left bundle branch block: randomised multi-centre clinical outcome study.

The His Optimised Pacing Evaluated for Heart Failure Trial

HOPE-HF

(15HH2828)

Study Investigators:

Chief Investigator: Dr Zachary Whinnett

Study Management: Imperial Clinical Trials Unit (ICTU)

SAP Working Group:

Emanuela Falaschetti – Senior Statistician

Nicholas Johnson – Study Statistician

Version	Date	Author
1.2	15th September 2020	Nicholas Johnson

Approved by:

Name	Signature	Role	Date
Dr Zachary Whinnett	Zachary Whinnett Digitally signed by Zachary Whinnett Date: 2020.09.17 10:05:04 +01'00'	Chief Investigator	
Prof. John Cleland		TSC Chair	Sep 17, 2020
Emanuela Falaschetti	Emanuela Falaschetti Digitally signed by Emanuela Falaschetti Date: 2020.09.17 09:19:45 +01'00'	Senior Statistician	
Nicholas Johnson	Nicholas A Johnson Digitally signed by Nicholas A Johnson Date: 2020.09.15 16:50:26 +01'00'	Study Statistician	

AV optimisation delivered with direct His bundle pacing, in patients with heart failure, long PR without left bundle branch block: randomised multi-centre clinical outcome study.

The His Optimised Pacing Evaluated for Heart Failure Trial - HOPE-HF

Table of Contents

2. Study Objectives / Hypotheses Testing 6
2.1. Primary Objectives 6
2.2. Secondary Objective 6
3. Study Endpoints 6
3.1. Primary Endpoint 6
3.2. Secondary Endpoints 6
3.3. Exploratory Endpoints 6
4. Background/Introduction 7
4.1. Introduction 7
4.2. Study Design 8
4.3. Treatment Groups 8
4.4. Study Population 8
4.5. Sample Size 9
4.6. Schedule of Time and Events 10
4.7. Randomisation and Crossover 12
4.8. Unblinding 12
4.9. Additional Analysis 12
4.9.1. Per-Protocol Analysis 12
4.9.2. Exploratory Analysis 12
4.9.3. Exploratory Sub-group Analysis 12
4.9.4. Sensitivity Testing 12
5. Analysis Sets 13
6. Variables of Analysis 13
6.1. Baseline Demographic Variables 13
6.2. Primary Endpoint Variable 14
6.3. Secondary Endpoint Variables 14
6.4. Exploratory Endpoint Variables 15
6.5. Variables for Exploratory Sub-group Analysis 15
6.6. Variables for Safety Analysis 16
7. Statistical Methodology 16
7.1. Baseline Demographics 16
7.2. Primary Analysis 16

7.3. Secondary Analysis 17
7.4. Per-Protocol Analysis 17
7.5. Exploratory Variable Analysis 17
7.6. Exploratory Sub-group Analysis 17
7.7. Missing Data and Imputation 19
7.8. Sensitivity Testing (in lieu of subgroup analysis) 19
7.9. Additional Sensitivity Testing 19
7.10. Safety Analysis 19
7.11. Interim Analysis 20
7.12. Protocol Deviations 20
7.13. Tables to Present 21

Changes from SAP v1.1 (31st July 2019)

Rationale –Due to the COVID-19 pandemic, the HOPE-HF study has had to be suspended. As a result, a few amendments have had to be made to account for a) the delay in last patient visit and b) any potential patient effect. An additional sub-group analysis investigating His-Pacing has also been included under recommendation from the TSC.

This amendment has also allowed for the daily activity data to be fully defined following the provision of dummy data from the device and has also made adjustments to the Per Protocol analysis set.

Section 3.3 – Additional section of exploratory endpoint variables defined to account for the additional variables contained in the external daily activity dataset.

Section 4.5 – Additional passage added to sample size to refer to the additional 8 patients recruited to cover the slight increase in missing data due to death, loss to follow-up, etc...

Section 4.9 – Collated 'additional analysis' section added for background info. Per Protocol analysis separated from sensitivity analysis as recommended by Morris et al (2014), Additional exploratory analysis as per Section 3.3 included, Sensitivity analysis section updated to specify an analysis to removed patients whose final visit was delayed by the COVID-19 pandemic.

Section 5 – Per protocol analysis set clarified.

Section 6.1 – Timepoint of baseline variables clarified.

Section 6.4 - Additional exploratory analysis variables as per Section 3.3 included.

Section 7.2 – Additional detail provided for primary analysis including SAS syntax.

Section 7.5 - Additional exploratory analysis as per Section 3.3 specified

Section 7.6 – Sub-group analysis models for Mean Daily Exercise Activity & Haemodynamic Response provided

Section 7.9 – Analysis method clarified for hospitalisation rates.

Changes from SAP v1.0 (18th October 2018)

Rationale –A series of edits and clarifications were requested after discussion with the TSC chair. The edits are minor in nature and do not change the primary endpoint or method of analysis. The majority of changes refer to the exploratory subgroup analysis but also cover sensitivity testing (per protocol & missing data analysis)

Section 3.2 - Secondary endpoint added for Haemodynamic Response

Sections 4.8, 6.4 & 7.4 - Content of sections revised to remove duplication of information and improve document layout

Section 4.8 – Clarification regarding the outcome variables to be used for the sub-group analysis. These are reduced to primary outcome, MVO2 and secondary endpoints daily activity and haemodynamic response.

Section 4.9 – Additional sensitivity analysis defined for primary and secondary analysis based on a per-protocol population

Section 5 – Additional per-protocol analysis population defined

Section 6.3 – Additional secondary endpoint variable for Haemodynamic Response added.

Section 6.3 – Clarification of timepoints for data collection included.

Section 6.4 - Clarification of timepoints for data collection included. Additional subgroup analysis for height defined

Section 7.2 – Extra details provided for primary analysis; fixed & random model variables defined, software/analysis package defined

Section 7.4 – Sub-group analysis section revised to incorporate above changes. Some dichotomized variables now revised to include tertile groupings.

Section 7.5 – Section for missing data and imputation has been added to specify the extent of missing data expected and to define a sensitivity analysis to explore missing data.

Section 7.6 – Additional sensitivity analysis added based on the newly defined per-protocol population

Section 7.7 – Mixed Effect Logistic Regression model analysis specified for event rates covering death, hospitalization and device-based issues

Section 7.8 – Additional passage covering checking of withdrawal rates in conjunction with the sample size added.

Section 7.10 – Tables, figures and listings updated to reflect the above changes.

2. Study Objectives / Hypotheses Testing

2.1. Primary Objectives

To evaluate whether, a group of heart failure patients who are not currently targeted for pacing therapy for heart failure, will obtain benefit from pacing therapy delivered to allow optimisation of LV preload by shortening Atrioventricular delay.

2.2. Secondary Objective

To assess direct His pacing characteristics in this population (including acute and chronic pacing thresholds).

3. Study Endpoints

3.1. Primary Endpoint

- The primary outcome will be exercise capacity measured using peak oxygen uptake (VO_2). To be measured at baseline and also at 6 and 12 months post randomisation.

3.2. Secondary Endpoints

- Echocardiographic measurement of left ventricular function and remodelling
- Changes in B-type Naturietic Peptide (BNP)
- Changes in daily activity levels (recorded using objective metrics from the pacemaker device)
- Changes in haemodynamic response
- Changes in Exercise Time (during MVO_2 testing)
- Changes in Quality of Life Scores
- Cost effectiveness analysis
- Percentage pacing will be recorded, as will arrhythmia burden (atrial fibrillation, atrial flutter and ventricular arrhythmias), pacing thresholds and lead impedance. Fluoroscopy time during device insertion will also be reported.

3.3. Exploratory Endpoints

Based on objective metrics recorded on the pacemaker device, we are able to explore the following additional endpoint measures

- Average Ventricular Rate per Day
- Average Ventricular Rate per Night
- Heart Rate Variability
- Daily Impedance

4. Background/Introduction

4.1. Introduction

In this study we will evaluate whether, a new group of heart failure patients who are not currently targeted for pacing therapy for heart failure, obtain benefit from pacing therapy delivered to allow optimisation of LV preload by shortening AV delay.

Our study involves two novel aspects. Firstly, in order to reduce the risk of causing harm by inducing ventricular electrical dyssychrony, we will use a special form of pacing – direct His bundle pacing- which has not previously evaluated in this context. Secondly, we will use a novel method for identifying optimal AV delay, which is designed to ensure precise and reproducible values are obtained.

His bundle pacing: a Medtronic Select Secure 3830 pacing lead is positioned at the His bundle using a 9 French delivery sheath (either the Medtronic C315 fixed shaped catheters or the Medtronic SelectSite C304 deflectable catheter). His capture is confirmed using the criteria previously described by Deshmukh et al. In brief these consist of confirming the following: a) 12 lead ECG morphology match with direct His bundle pacing compared with intrinsic conduction, b) similar time delay between the stimulation artefact and the onset of the QRS complex compared with the intrinsic His to QRS time, c) His bundle capture in an all or none-fashion, demonstrated by the absence of QRS widening at a lower pacing output.

If selective His bundle pacing cannot be achieved then non-selective His bundle pacing will be accepted. Non-selective His pacing is defined as the direct capture of the basal ventricular myocardium in addition to His bundle capture.

AV delay optimisation: will be performed using acute non-invasive blood pressure acquired using the Finometer device (Finapres Medical systems, Netherlands). The BHF alternation protocol we have previously described will be used in order to minimise the effect of background noise.

The optimal AV delay will be determined during atrial sensing and atrial pacing. For atrial pacing the optimisation will be performed at 10-20 bpm above the resting heart rate provided AV conduction is 1:1 during AAI pacing at this heart rate. If Wenkebach conduction or heart block occurs at this rate then we will lower heart rate to in order to achieve 1:1 conduction. If conduction is not 1:1 at the lower heart rate then the paced AV delay optimisation will be performed with a reference setting of His pacing with an AV delay of 120ms.

For atrial sensing, optimisation will be performed at resting heart rate.

Patients are randomised to 6 months of the following:

1) Active treatment: His bundle pacing with the AV delay programmed to the optimal sensed and paced delays identified during the optimisation process.

2) No His pacing treatment: pacemaker programmed to avoid pacing as per the implanting physicians discretion utilising the non-His ventricular lead (suggested modes are VVI 30, DDD/I 30 etc). His pacing should only be programmed on active treatment.

Dynamic AV delay will be programmed off throughout the study.

4.2. Study Design

This is a multi-centre, prospective randomised double-blinded cross over study, recruiting a sub-population of patients with heart failure from multiple investigational sites in the UK. Recruitment is expected to take place over about 2 years.

All patients are being implanted with a Pacemaker or Implantable Cardioverter Defibrillator with one of the leads positioned on the His bundle in order to obtain His-bundle capture. There will be a 2-month run-in period where the device will be programmed not to deliver His pacing therapy during this period.

Patients are allocated in random order to six month treatment periods in each of the following two states (1) No His bundle pacing; (2) AV optimised His-bundle pacing. Endpoint measurements will be taken at baseline, 6 months and 12 months post randomisation. Treatment allocation will be blinded to the endpoint assessor.

4.3. Treatment Groups

Table 1: Summary of Treatment Groups

Treatment Arm	Period 1 (M0 –M6)	Period 2 (M6 –M12)
1	Active Pacing	Back-up Only (No) Pacing
2	Back-up Only (No) Pacing	Active Pacing

4.4. Study Population

The HOPE-HF study is recruiting patients aged 18 or above that have a Ventricular Ejection Fraction (EF) of 35% and below (based upon an echocardiograph reading taken no more than 18 months from screening). In the event of a patient having an EF between 35% and 40%, inclusion can still take place on the condition that BNP has to be greater than 250 ng/L. The patient is also required to have a PR interval greater than or equal to 200ms and a narrow QRS duration (≤140ms) or prolonged QRS duration with typical Right Bundle Branch Block (RBBB) morphology on 12 lead ECG and sinus rhythm (based upon an ECG reading taken no more than 18 months from screening). All patients are required to display symptoms that fit within classes II – IV of the New York Heart Association (NYHA).

In order to be eligible for the study the patient must not have any of the following:

- Permanent or persistent atrial fibrillation (AF)
- Paroxysmal atrial fibrillation with history of sustained AF (more than 24 hours) in the 6 months prior to screening
- Patients who are unable to perform cardiopulmonary exercise testing
- Other serious medical condition with life expectancy of less than 1 year
- Lack of capacity to consent
- Pregnancy (female participants of reproductive age will be eligible for inclusion in the study, subject to a negative pregnancy test prior to randomisation)

- Contraindication to use of the relevant study device or leads (as per current manuals from manufacturer)

Eligible patients can be sub-divided into 2 groups:

A. patients who do not have an indication for an implantable ICD;
B. patients who do have an indication for an implantable ICD.

All eligible patients will then attend for implantation of their device. In patients who do not have an indication for an ICD a second ventricular lead will be implanted in a lateral branch of the coronary sinus. If direct His pacing has not been successfully achieved, then a further lead will be positioned at the RV apex. In patients who do have an indication for an ICD, the ICD lead will be positioned in the right ventricle (either RV apex or RV septum).

Randomisation will occur only after a 2 month run-in period has been completed.

4.5. Sample Size

A smoothed modified Bruce protocol for patients with heart failure is used. This enables improved reproducibility of peak VO_2. With this approach, reproducibility in heart failure patients between two separate visits to the hospital is 2.4 ml/kg/min, expressed as the standard deviation of difference between two independent measurements. Implantation of a cardiac resynchronisation therapy pacemaker in patients with broad QRS duration and heart failure results in a 0.5-2.5 (mean 1.5) ml/kg/min increase in peak $V0_2$. Our published pilot data is that switching on classical cardiac resynchronisation therapy in patients with a broad QRS duration increments LV systolic blood pressure by ~10.3mmHg, and switching on direct His-bundle pacing in long-PR/narrow-QRS patients increments blood pressure by ~6.3mmHg, i.e. ~60% of that effect. Pro-rata we would therefore expect an estimated increment in peak VO2 of 60% of 1.5ml/kg/min, i.e. 0.9 ml/kg/min.

Adopting a conservative approach, a two-sided alpha of 0.05 is chosen. Using a paired t-test, in order to detect a difference of 0.7 with SD of 2.4, to achieve a power of 90% would require a total of 126 evaluable patients. Allowing for a combined mortality/dropout of 21%, the study would require a total sample size of 160 patients. Consequently, 160 patients will be initially randomised in a 1:1 manner to the treatment groups.

Following a review on data completeness on November 2018 there was evidence to suggest that the mortality/dropout rate was higher than the 21% originally stated. To ensure the study was adequately powered an additional 7 patients were randomised into the study (based on 188 patients being enrolled, 172 proceeding to implant).

4.6. Schedule of Time and Events

Table 2: Treatment Regimens

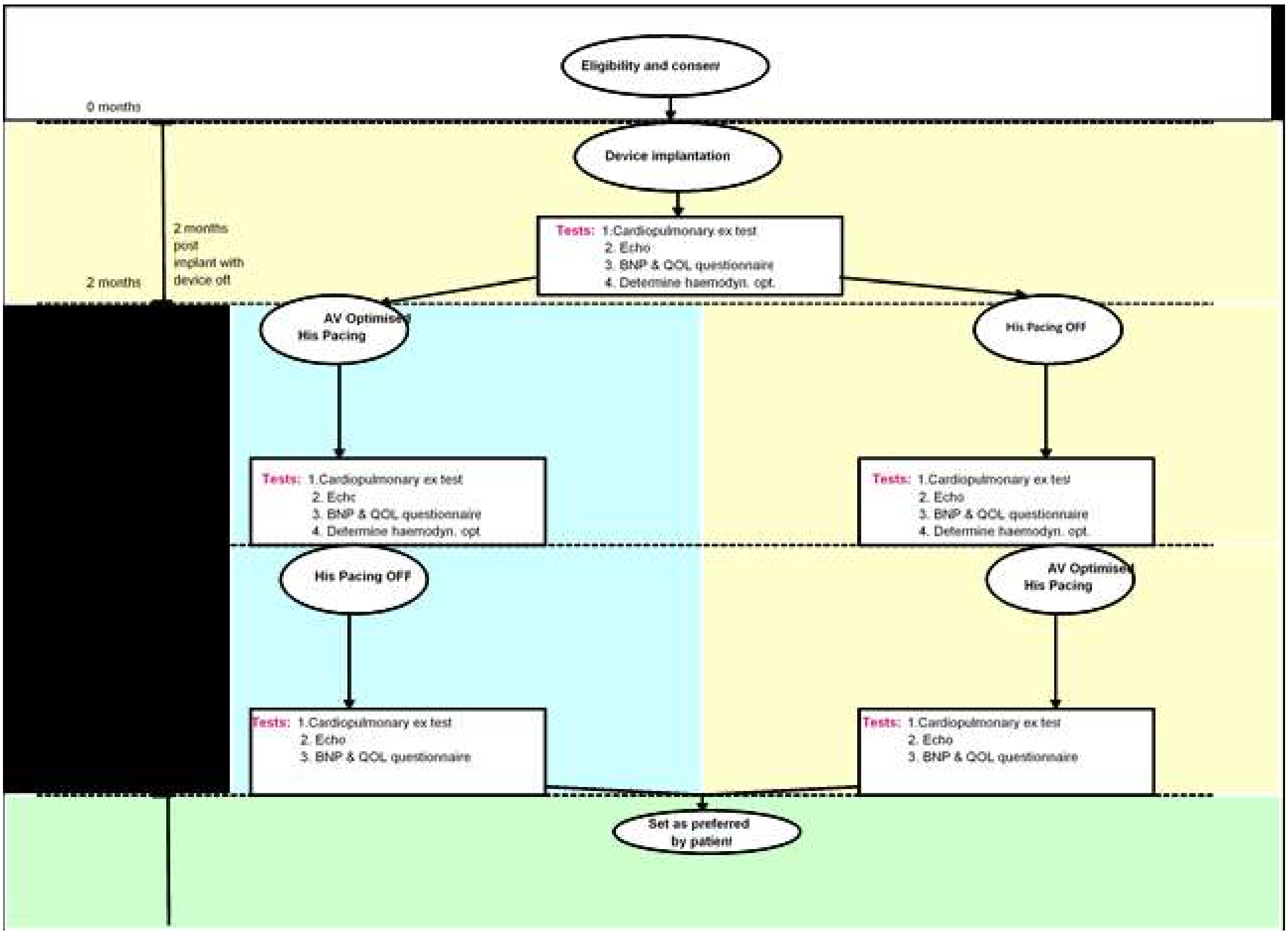

Table 3: Summary of study procedures/assessments by visit

‡ Visit at local hospital where patient was identified
^ Performed at Imperial College Healthcare NHS Trust
& Taken at screening visit or within the past 18 months
* RUM questionnaire only at visit 1, all questionnaires for visits 3-5

Either at local hospital or at Imperial College Healthcare NHS Trust
£ Taken on the day or within the last 12 months
$ Taken on the day of implantation or within the past 30 days

	**Screening‡**	**Implantation#**	**Randomisation**	**Cross-over**	**End of Study**
**Visit**	**1**	**2**	**3**	**4**	**5**
Month/Week/Day	- 2 to 6 months	-2 months (± 14 days)	0	6 months (± 14 days)	12 months (± 14 days)
Informed consent	X				
Inclusion & exclusion criteria	X				
Demography	X				
Medical history	X				
Vital signs	X	X	X	X	X
Concomitant medication	X				
Pregnancy test for women of child bearing age	X				
Safety blood tests (Creatinine, Haemoglobin, PT, G+S)		X£			
ECG	X&	X$			
Device Implantation		X			
Post implantation chest radiograph		X			
Pacing check			X	X	X
Device programmed on/off			X	X	X
CPET			X	X	X
Echocardiography			X	X	X
BNP			X	X	X
QOL and Cost Effectiveness questionnaires (EQ5D-5L/MLHFQ and the HOPE-HF RUM)	X*		X	X	X
Haemodynamic optimisation			X	X	X
Revelation of treatment sequence to patient					X
His pacing on/off according to patient's choice (Patient can make decision later)					X

4.7. Randomisation and Crossover

Two months after patients are implanted with their device, patients are randomised to either receive active pacing treatment or back up only pacing (pacemaker programmed to VVI 30 bpm). After a further 6 months they are crossed over to the alternative treatment arm. Treatment allocation is obtained using an Interactive Web Response System (IWRS) programmed with a randomisation schedule provided by the trial statistician with use of appropriate blocking.

The patient has study assessments as per Section 4.6 above.

The physiologists performing study tests will be blinded to the treatment arm. A separate investigator will be responsible for programming the device.

4.8. Unblinding

Patients are blinded to treatment arm. We anticipate that some patients may request a change in their treatment arm, particularly if there is a change in their symptom status. If this occurs we will try to encourage patients to continue with their designated treatment (either pacing or no pacing) until they cross over to the other treatment of complete the study. However, if they are not willing to continue treatment then their request for change in treatment will be met.

The master randomisation list will be stored electronically within a specialised 'blinded' folder in the statistics section of the Trial Master File (TMF) to ensure that the trial statistician does not have access to the list. A copy will also be made available to dedicated personnel in the research unit who will support unblinding of individual patient's treatment allocation upon request. Requests for unblinding should be discussed with the Chief Investigator or delegate beforehand.

Once unblinded, all summary tables and listings produced for the trial report will show the allocated treatment arm, treatment period and corresponding treatment for that period.

4.9. Additional Analysis

4.9.1. Secondary Per-Protocol Analysis

To investigate the potential effect on the final analysis of those patients that failed to adhere to the study protocol, an additional per-protocol analysis will be run on the primary and secondary outcome measures using a Per-Protocol population as defined in section 5.

4.9.2. Exploratory Analysis

We will perform analysis to explore if there is a different treatment effect on data provided by the pacemaker device as listed within Section 3.3.

4.9.3. Exploratory Sub-group Analysis

We will perform analysis to explore if there is a different treatment effect on peak VO2 and additional exploratory endpoints (daily activity and haemodynamic response) by the subgroup variables defined in Section 6.4.

4.9.4. Sensitivity Testing

In some circumstances sub-group analysis may not be feasible due to a sub-group arm being under-populated. In the event where a sub-group analysis is deemed unsuitable, sensitivity testing will be used instead. The sensitivity analysis will exclude patients from the under-populated sub-group arm thus assessing whether the inclusion of these patients had any bearing on the results

of the original analysis. One sensitivity analysis will exclude the patients who had their final visit delayed (originally due March/April 2020) due to the COVID-19 pandemic.

5. Analysis Sets

All Primary & Secondary analysis will be performed using data collected from all randomised subjects as according to the intention to treat principle.

Sub-group analysis (as per section 4.11) will use the same set of data collected from randomised patients (as above). Where subject data is unavailable for a sub-group variable of interest the subject will be excluded from that sub-group analysis

The population used for additional (secondary) per-protocol testing of primary and secondary outcome measures (as per sections 6.2 & 6.3) will take the ITT population and remove the following:

- Any patients which fail to attend follow-up visits (within the specified windows) at 6 months or 12 months).
- Any patients which have treatment terminated early during either 6 month period.
- Any patients with LV-lead implanted instead of HIS lead.
- Any patients with low ventricular pacing (defined as less than 90%) at Randomisation.
- Any patients that record a raised His-capture threshold (above 3V). fail to receive pacing successfully during the allocated pacing treatment phase *

  * Failure to receive pacing will be assessed by the clinical team reviewing data including threshold raises (His-capture threshold (above 3V) and other data collected at the device check. To account for any potential bias the review should be undertaken whilst the clinical team is still blinded. A sensitivity analysis should be run alongside the per-protocol analysis including patients removed under these criteria to evaluate whether clinician-removed data effected the per-protocol outcomes.

6. Variables of Analysis

6.1. Baseline Demographic Variables

The following variables will be summarised as baseline demographic variables. The subject baseline visit is to be defined as visit 3 (randomisation). In the event where the subject does not have data available for that particular visit, baseline data will be taken from the closest visit preceding visit 3 (randomisation). where data is available :

- Age (taken at screening)
- Ethnicity (taken at screening)
- Gender (taken at screening)
- Vitals (Height, Weight, BMI, Average Systolic/Diastolic Blood Pressure)
- MVO_2 (Peak VO_2)
- NYHA Class
- Past HF aetiology (taken at screening)

6.2. Primary Endpoint Variable

Peak oxygen intake (MVO_2) at BL, 6 Months and 12 Months

6.3. Secondary Endpoint Variables

The following variables will be analysed as secondary objective measures and measured at BL, 6 Months and 12 Months:

- Left ventricular end-diastolic diameter
- Left ventricular end-systolic diameter
- Left ventricular end-diastolic volume
- Left ventricular ejection fraction
- B-type Naturietic Peptide (BNP)
- Exercise Time (during MVO_2 testing)
- Daily activity time *
- ED5Q5L Score
- Minnesota Questionnaire Score
- AF/SVT burden
- Haemodynamic Response *

* Daily activity is defined as the mean daily activity over the treatment period and will be recorded using objective metrics from the pacemaker device and will be stored outside of InForm. Haemodynamic response is defined as the magnitude of change in mmHg blood pressure during optimisation (under both sensed and paced AV delay).

The following secondary objective measures will be presented via descriptive statistics:

- Impedance of RA lead
- Impedance of His lead
- Impedance of LV/ICD lead
- Pacing threshold RA lead at 0.4mV
- Pacing threshold His Lead, His capture threshold
- Pacing threshold His lead: lowest capture threshold (stored outside of InForm)
- Pacing threshold LV/ICD lead at 0.4mV
- Sensed P wave amplitude
- Total fluoroscopy time (at device insertion)

Cost effectiveness analysis will be performed by a Health Economist. The variables of analysis required will be specified in a separate plan external to the SAP.

6.4. Exploratory Endpoint Variables

The following additional metrics have been made available from data recorded on the device. These will be provided in the form of daily data recorded over the 12 month treatment period and overall mean per 6-month period.

- Average Ventricular Rate per Day
- Average Ventricular Rate per Night
- Heart Rate Variability
- Daily Impedance

6.5. Variables for Exploratory Sub-group Analysis

In order to investigate the additional sub-group analyses referred within section 4.8 the following variables will be assessed against peak VO2 and additional endpoints; daily activity and haemodynamic response* at visits 3 (randomisation), 4 (cross-over) and 5 (end of follow-up):

1. QRS Morphology at Insertion (V2)
2. PR Interval at Insertion (V2)
3. Baseline Echocardiography defined by E/A Fusion (recorded outside of InForm) at Insertion (V2)
4. Selective vs Non-Selective His Bundle Pacing at Insertion (V2)
5. LV Lead vs His Lead at Insertion (V2)
6. Ejection Fraction Group at Randomisation (V3)
7. Device Type (ICD vs non-ICD) at Insertion (V2)
8. BMI at Randomisation (V3)
9. Max RER Score at Randomisation (V3)
10. Height at Screening (V1)
11. Percentage His-pacing at Randomisation (V3)
12. Adherence to optimal pacing based on his-capture metrics obtained at V4/V5 and AE data [Ͳ].

* Daily activity is defined as the mean daily activity over the treatment period and will be recorded using objective metrics from the pacemaker device and will be stored outside of InForm. Haemodynamic response is defined as the magnitude of change in mmHg blood pressure during optimisation (under both sensed and paced AV delay).

[Ͳ] This will be established on a per-patient basis using blinded data and will be dependent on raises in threshold and other metrics derived from device check data. Patients with events affecting treatment (for example, AF) will also be placed in the ‘poor’ adherence subgroup.

6.6. Variables for Safety Analysis

To assess patient and study safety the following variables will be recorded and analysed:

- Adverse events / Serious Adverse events (Including Lead Displacement, Hospitalisation & Death)
  - Rate of Death
  - Rate of Hospitalisation
- Adverse Device Effects / (Unanticipated and Anticipated) Serious Adverse Device Effects
  - Revision of Device (Replacement, Repositioning)
  - Extraction/Removal of Device (due to infection etc…)
- Vital Signs (Weight, Pulse, BMI, Ave. Systolic/Diastolic Blood Pressure)

7. Statistical Methodology

7.1. Baseline Demographics

Patient characteristics will be summarized. Frequency tables with percentages will be produced for categorical data and output for continuous data will present the mean, standard deviation, median, inter-quartile range, minimum & maximum.

7.2. Primary Analysis

The primary outcome will be analysed using a two-level hierarchal model. We will include treatment, period and sequence effect within the model as fixed effects. A random effect for subject will also be included in the regression model and will take the following form:

$$Y_{iv} = \mu + \tau_{iv} + \pi_{iv} + s_i + b_i(s_i) + e_{iv}$$

Let MVO_{2iv} represent peak VO_2 for patient i at visit v (V4 or V5). We will model MVO_{2iv} as the sum of five components:

MVO_{2iv} = intercept + treatment_{iv} + period_{iv} + sequence_i + $\text{patient}_i(\text{sequence}_i)$ + residual error_{iv}

- intercept term (fixed effect): represents peak VO_2 under no pacing
- treatment term: the primary outcome, represents the difference in MVO_2 between Pacing and No Pacing
- period term: represents the period of treatment (period 1 – between V3 & V4; period 2 – between V4 & V5)
- sequence term: the arm represents the sequence effect (arm 1 or 2)
- patient(sequence) term: the random effect for patient *i* within their allocated sequence
- (residual error term is assumed to have a normal distribution)

The normality of MVO_2 will be checked and an appropriate transformation will be undertaken prior to analysis if necessary. If normality cannot be achieved via transformation, non-parametric methods will be used. Analysis will be carried out using PROC MIXED in SAS or using the corresponding command in an alternative software package.

```
PROC MIXED DATA=FinalAnalysisData METHOD=ML;
  CLASS patient treatment period sequence;
  MODEL MVO2 = treatment period sequence /s CL ;
  RANDOM PATIENT(SEQUENCE);
run;
```

Statistical testing will be two-tailed with a 5% significance level. The estimated solution for the treatment fixed-effect presented alongside the 95% confidence interval and subsequent p-value. Any other significant terms in the model will also be reported.

Endpoint values will also be presented in a summary table by arm, treatment and visit. A corresponding listing will also be provided as part of an appendix.

7.3. Secondary Analysis

Differences in secondary outcomes will also be analysed using the hierarchal model described above in Section 7.2. Normality will be checked and appropriate transformation or non-parametric methods will be used as appropriate.

All statistical tests will be two-tailed with a 5% significance level with the estimated solution for the treatment fixed-effect presented alongside the 95% confidence interval and subsequent p-value. Any other significant terms in the model will also be reported.

Cost effectiveness analysis will be performed by a Health Economist. The method of analysis will be specified in a separate plan external to the SAP.

7.4. Per-Protocol Analysis

The primary and secondary analysis discussed in section 7.2 & 7.3 above will be re-run as additional secondary analysis using the Per-Protocol population as defined in section 5.

7.5. Exploratory Variable Analysis

Differences in exploratory outcomes will also be analysed using the hierarchal model described above in Section 7.2. Normality will be checked and appropriate transformation or non-parametric methods will be used if it is not met.

No formal statistical testing will be carried out. The estimated treatment effect from the hierarchal model will be presented alongside its corresponding 95% confidence interval.

7.6. Exploratory Sub-group Analysis

For the sub-group analysis variables defined within Section 6.5, each will be individually added to the hierarchal model described within Section 7.2 (along with the corresponding interaction term investigating the sub-group variable against treatment). The sub-group model for MVO_{2iv} (peak VO_2 for patient i at visit v) will be as follows:

$$MVO_{2iv} = intercept_i + treatment_{iv} + \textit{subgroup\_var}_i + treatment_{iv} * \textit{subgroup\_var}_i + period_{iv} + sequence_i + residual\ error_{iv}$$

The above term *subgroup_var*$_i$ will represent the effect of the following sub-group variables as per Section 6.5:

<u>Continuous</u>
1. PR Interval
2. BMI
3. Height
4. Percentage His-pacing

<u>Categorical</u>
1. QRS Morphology based on QRS category (RBBB, LBBB, Narrow QRS Duration, Other)

<u>Ordinal</u>
1. Max RER Score (Three groups: <0.95, 0.95-1.05, >1.05)

<u>Binary</u>
1. Ejection Fraction Group (Dichotomised as ≤35% vs >35%) at insertion
2. Baseline Echocardiography (E/A Fusion - yes vs no)
3. Selective vs Non-Selective His Bundle Pacing
4. Device Type (ICD vs non-ICD)
5. LV Lead vs His Lead
6. Adherence to optimal pacing (yes vs no) based on metrics obtained at V4/V5 and AE data

Corresponding models will also be produced for haemodynamic response and daily activity:

The sub-group model for Daily Activity$_{iv}$ (mean recorded activity for patient i over visit v) will be as follows:

$$D\_Activity_{iv} = intercept + treatment_{iv} + \textit{subgroup\_var}_i + treatment_{iv} * \textit{subgroup\_var}_i + period_{iv} + sequence_i + patient_i(sequence_i) + residual\ error_{iv}$$

The sub-group model for Haemodynamic Response$_{iv}$ (peak VO_2 for patient i at visit v) will be as follows:

$$H\_Resp_{iv} = intercept + treatment_{iv} + \textit{subgroup\_var}_i + treatment_{iv} * \textit{subgroup\_var}_i + period_{iv} + sequence_i + patient_i(sequence_i) + residual\ error_{iv}$$

Alongside the output from testing the above hierarchal model, results will be presented in a summary table by subgroup, arm, treatment and visit. Median values will be presented in summary tables.

Subgroup analyses will be collated and presented in a forest plot for comparison. To achieve this, any continuous variables will be dichotomised or presented as tertiles using a reference standard. Dichotomisation will take place at the median for normally-distributed variables unless specified. Likewise, boundaries for tertiles will take place at the 33.3rd and 66.7th percentiles for normally-distributed variables unless specified (see example forest plot 6.11.1 – 6.11.3 on page 31).

No formal statistical testing will be carried out. The estimated treatment effect from the hierarchal model will be presented alongside its corresponding 95% confidence interval.

7.7. Missing Data and Imputation

The mixed-model for the primary, secondary and subgroup analysis will incorporate all subject data for MVO2 collected between visits 3 and 5 such that patients with non-complete profiles will still be included. In incorporating a random effect for inter-subject variability, under the assumption of data missing-at-random (MAR), the analysis model is equipped to cope with missing data for subjects with incomplete data profiles. On the basis of this and confirmation that the withdrawal rate is not exceeding expectations, no imputation for missing data will take place for the primary analysis.

A sensitivity analysis will be run alongside the primary analysis to investigate the potential effect the missing data may have on the results of the primary analysis. Based on subject arm and treatment period, data will be imputed based on the highest and lowest values provided in that period for that particular treatment arm. Analysis will be re-run with both the high and low imputed values and any change in results will be reported.

7.8. Sensitivity Testing (in lieu of subgroup analysis)

Under the circumstance where a particular sub-group analysis population is too low to run any of the sub-group analyses defined in Section 4.8 a sensitivity analysis will be run in its place. To achieve this the primary analysis will be re-run excluding patients from the under-populated sub-group arm thus assessing whether the inclusion of these patients had any bearing on the results of the original analysis.

7.9. Additional Sensitivity Testing

Outside of sensitivity testing where subgroup sizes are too small. Additional testing may be carried out to investigate potential scerarions where data might be affected. For example, sensitivity analysis on the primary/secondary outcomes will be carried out to exclude the patients who had their final visit delayed (originally due March/April 2020) due to the COVID-19 pandemic.

7.10. Safety Analysis

Adverse events and adverse device effects will be summarised by arm, treatment and severity. A separate table summarising adverse events and their relationship to study treatment will also be produced.

Serious adverse events and serious adverse device effects will be listed and summarised by site, SAE category, arm and treatment. A separate table summarising serious adverse events and their relationship to study treatment will also be produced.

All other safety variables will be summarized by treatment and timepoint in the form of frequency tables for categorical variables or descriptive statistics for continuous variables.

Rates of Death, Revision of Device (Replacement, Repositioning) and Extraction/Removal of Device (due to infection etc…) will be assessed using a mixed-effects logistic regression model and will include fixed effect variables for treatment, period and sequence and a random effect variable for subject. Hospitalisation rates will be assessed using a mixed-effects Poisson regression model.

7.11. Interim Analysis

Interim reports detailing baseline demographics, safety variables and the primary endpoint (if requested) will be produced for assessment and discussion at DMEC meetings, the framework of which will be provided within the DMEC charter.

At 50% randomisation (80 patients) and 75% randomisation (120 patients) a review of retention and withdrawal rates from Visit 1 (enrolment) to Visit 3 (randomisation) and from Visit 3 to Visit 5 (end of follow-up) was undertaken to ensure enough patients were enrolled, implanted and randomised to ensure the target of 126 evaluable patients were met.

Other than the above, no formal interim analyses are planned.

7.12. Protocol Deviations

Protocol deviations are to be listed and summarised by category and site.

7.13. Tables to Present

Table 1.1: Subject Disposition

	Site A	Site B	…	Total
**Screened**				
**Randomised**				
**Arm A**				
**Arm B**				
**Withdrawn**				
Reason for Withdrawal				
**Completed**				

Table 2.1: Number of protocol deviations by centre and category

Type of Deviation	Site A	Site B	…	Total
Patient was incorrectly included in the trial (did not meet all the inclusion and exclusion criteria				
⋮				
Patient pregnancy				
Other				
**Total**				

Table 3.1: Summary of Baseline Demographics

Variable	Statistics	Arm A [N=XX]	Arm B [N=XX]	All Subjects [N=XX]
Age (y)	n			
	Mean (SD)			
	Median (IQR)			
	Min, Max			
Ethnicity (%)	Asian			
	Black			
	Mixed			
	White			
Gender (%)	Female			
	Male			
Height (cm)	n			
	…			
Weight (kg)	n			
	…			
BMI	n			
	…			
Pulse	n			
	…			
Systolic BP	n			
	…			
Diastolic BP	n			
	…			

Table 4.1: Summary of MVO2 values by treatment and visit

Arm	Visit	Treatment	n	Mean	SD	Median	IQR	Min.	Max.
A	BL	-							
	M6	No-Pacing							
	M12	Pacing							
B	BL	-							
	M6	Pacing							
	M12	No-Pacing							

Table 4.2: Output of mixed-model test results for MVO2 by arm, treatment and timepoint

Type 3 Tests of Fixed Effects				
**Effect**	**Num DF**	**Den DF**	**F Value**	**Pr > F**
**Treatment**				
**Period**				
**Arm**				
**xxxx**				
**(Interaction Terms)**				

Solution for Fixed Effects									
**Effect**	**Treatment**	**Period**	**Arm**	**xxxx**	**Estimate**	**SE**	**DF**	**t Value**	**Pr > |t|**
**Intercept**			**-**						
**Treatment**	**…**		**-**						
	**…**		**-**						
**Period**		**…**	**-**						
		**…**							
**ARM**	**-**	**-**	**…**	**-**					
	**-**	**-**	**…**	**-**					
**xxxx**				**…**					
**(Interactions)**	**…**	**…**							

Least Squares Means							
Effect	Estimate	Standard Error	DF	t Value	Pr > |t|	Adjustment	Adj P

Table 5.1.1: Summary of Echocardiographic Variables

Variable*	Arm	Visit	Treatment	n	Mean	SD	Median	IQR	Min.	Max.
	A	BL	-							
		M6	No-Pacing							
		M12	Pacing							
	B	BL	-							
		M6	Pacing							
		M12	No-Pacing							

***Variables to assess: LV end-diastolic diameter & volume, LV end-systolic diameter, LVEF and LVOT diameter.**

Table 5.1.2: Output of mixed-model for Echocardiographic Variables by arm, treatment and timepoint

Please refer to series of output in Table 4.2. Tests to be carried out on the following variables: LV end-diastolic diameter & volume, LV end-systolic diameter, LVEF and LVOT diameter.

Table 5.2.1: Summary of BNP Values

Arm	Visit	Treatment	Samples Taken	n	Mean	SD	Median	IQR	Min.	Max.
A	BL	-								
	M6	No-Pacing								
	M12	Pacing								
B	BL	-								
	M6	Pacing								
	M12	No-Pacing								

Table 5.2.2: Output of mixed-model for BNP by arm, treatment and timepoint

Please refer to series of output in Table 4.2.

Table 5.3.1: Summary of Quality of Life Scores

Variable*	Arm	Visit	Treatment	n	Mean	SD	Median	IQR	Min.	Max.
	A	BL	-							
		M6	No-Pacing							
		M12	Pacing							
	B	BL	-							
		M6	Pacing							
		M12	No-Pacing							

***Variables to assess: ED5Q5L and Minnesota Scores**

Table 5.3.2: Output of mixed-model for Quality of Life Scores by arm, treatment and timepoint

Please refer to series of output in Table 4.2. Variables to assess: ED5Q5L and Minnesota Scores

Table 5.4.1: Summary of Device Check Variables

Variable*	Arm	Visit	Treatment	n	Mean	SD	Median	IQR	Min.	Max.
	A	BL	-							
		M6	No-Pacing							
		M12	Pacing							
	B	BL	-							
		M6	Pacing							
		M12	No-Pacing							

***Variables to assess: %age HIS Paced, AF/SVT Burden, Impedance of RA, His & LV/ICD Leads, Pacing Thresholds of RA, His & LV/ICD Leads, P-Wave Amplitude, Total Fluoroscopy Time**

Table 5.4.2: Output of mixed-model for Device Check Variables by arm, treatment and timepoint

Please refer to series of output in Table 4.2. Variables to assess: %age HIS Paced, AF/SVT Burden. Remaining variables are to be summarised only.

Table 5.5.1: Summary of Exercise Time values

Variable*	Arm	Visit	Treatment	n	Mean	SD	Median	IQR	Min.	Max.
	A	BL	-							
		M6	No-Pacing							
		M12	Pacing							
	B	BL	-							
		M6	Pacing							
		M12	No-Pacing							

Table 5.5.2: Output of mixed-model for Exercise Time values by arm, treatment and timepoint

Please refer to series of output in Table 4.2.

Table 5.6.1: Summary of Haemodynamic Response values

Variable*	Arm	Visit	Treatment	n	Mean	SD	Median	IQR	Min.	Max.
	A	BL	-							
		M6	No-Pacing							
		M12	Pacing							
	B	BL	-							
		M6	Pacing							
		M12	No-Pacing							

Table 5.6.2: Output of mixed-model for Haemodynamic Response values by arm, treatment and timepoint

Please refer to series of output in Table 4.2.

Sub-Group Analysis Output

Table 6.1.1 –6.1.3 - Output of mixed-model for MVO2/Daily Activity/Haemodynamic Response by arm, treatment and timepoint including sub-group variable for QRS Category

Please refer to output within Table 4.2

Table 6.1.4 –6.1.6 - Output of mixed-model for MVO2/Daily Activity/Haemodynamic Response by arm, treatment and timepoint including sub-group variable for QRS Category dichotomised as Narrow QRS vs Non-Narrow QRS

Please refer to output within Table 4.2

Table 6.2.1 –6.2.3 - Output of mixed-model for MVO2/Daily Activity/Haemodynamic Response by arm, treatment and timepoint including sub-group variable for PR Interval at Implantation (as continuous variable)

Please refer to output within Table 4.2

Table 6.2.4 –6.2.6 - Output of mixed-model for MVO2/Daily Activity/Haemodynamic Response by arm, treatment and timepoint including sub-group variable for PR Interval at Implantation (as tertile groups)

Please refer to output within Table 4.2

Table 6.3.1- 6.3.3 - Output of mixed-model for MVO2/Daily Activity/Haemodynamic Response by arm, treatment and timepoint including sub-group variable for patients grouped by E-A fusion

Please refer to output within Table 4.2

Table 6.4.1 –6.4.3 - Output of mixed-model for MVO2/Daily Activity/Haemodynamic Response by arm, treatment and timepoint including sub-group variable for selective vs non-selective His capture

Please refer to output within Table 4.2

Table 6.5.1 –6.5.3 - Output of mixed-model for MVO2/Daily Activity/Haemodynamic Response by arm, treatment and timepoint including sub-group variable for patients based on lead implantation (LV Lead vs His Lead)

Please refer to output within Table 4.2

Table 6.6.1 –6.6.3 - Output of mixed-model for MVO2/Daily Activity/Haemodynamic Response by arm, treatment and timepoint including sub-group variable for patients dichotomised by Ejection Fraction

Please refer to output within Table 4.2

Table 6.7.1 –6.7.3 - Output of mixed-model for MVO2/Daily Activity/Haemodynamic Response by arm, treatment and timepoint including sub-group variable for patients dichotomised by Device Type

Please refer to output within Table 4.2

Table 6.8.1 –6.8.3 - Output of mixed-model for MVO2/Daily Activity/Haemodynamic Response by arm, treatment and timepoint including sub-group variable for patients BMI (as continuous variable)

Please refer to output within Table 4.2

Table 6.8.4 –6.8.6 - Output of mixed-model for MVO2/Daily Activity/Haemodynamic Response by arm, treatment and timepoint including sub-group variable for patients BMI (as tertile groups)

Please refer to output within Table 4.2

Table 6.9.1 –6.9.3 - Output of mixed-model for MVO2 by arm, treatment and timepoint including sub-group variable for patients Max RER Value (as continuous variable)

Please refer to output within Table 4.2

Table 6.9.4 –6.9.6 - Output of mixed-model for MVO2 by arm, treatment and timepoint including sub-group variable for patients dichotomised by [illegible]

Please refer to output within Table 4.2

Table 6.10.1 –6.10.3 - Output of mixed-model for MVO2/Daily Activity/Haemodynamic Response by arm, treatment and timepoint including sub-group variable for patients height (as continuous variable)

Please refer to output within Table 4.2

Table 6.10.4 –6.10.6 - Output of mixed-model for MVO2/Daily Activity/Haemodynamic Response by arm, treatment and timepoint including sub-group variable for patients height (as tertile groups)

Please refer to output within Table 4.2

Table 6.11.1 –6.11.3 - Output of mixed-model for MVO2/Daily Activity/Haemodynamic Response by arm, treatment and timepoint including sub-group variable for percentage-His pacing (as continuous variable)

Please refer to output within Table 4.2

Table 6.11.4 –6.11.6 - Output of mixed-model for MVO2/Daily Activity/Haemodynamic Response by arm, treatment and timepoint including sub-group variable for percentage-His pacing (dichotomised) as ≤95% vs >95%

Please refer to output within Table 4.2

Table 6.12.1 –6.12.3 - Output of mixed-model for MVO2/Daily Activity/Haemodynamic Response by arm, treatment and timepoint including sub-group variable for His-Pacing adherence (as binomial variable)

Please refer to output within Table 4.2

Table 6.11.1 –6.11.3: Forest Plot of Sub-Group Analysis for MVO2/Daily Activity/Haemodynamic Response *

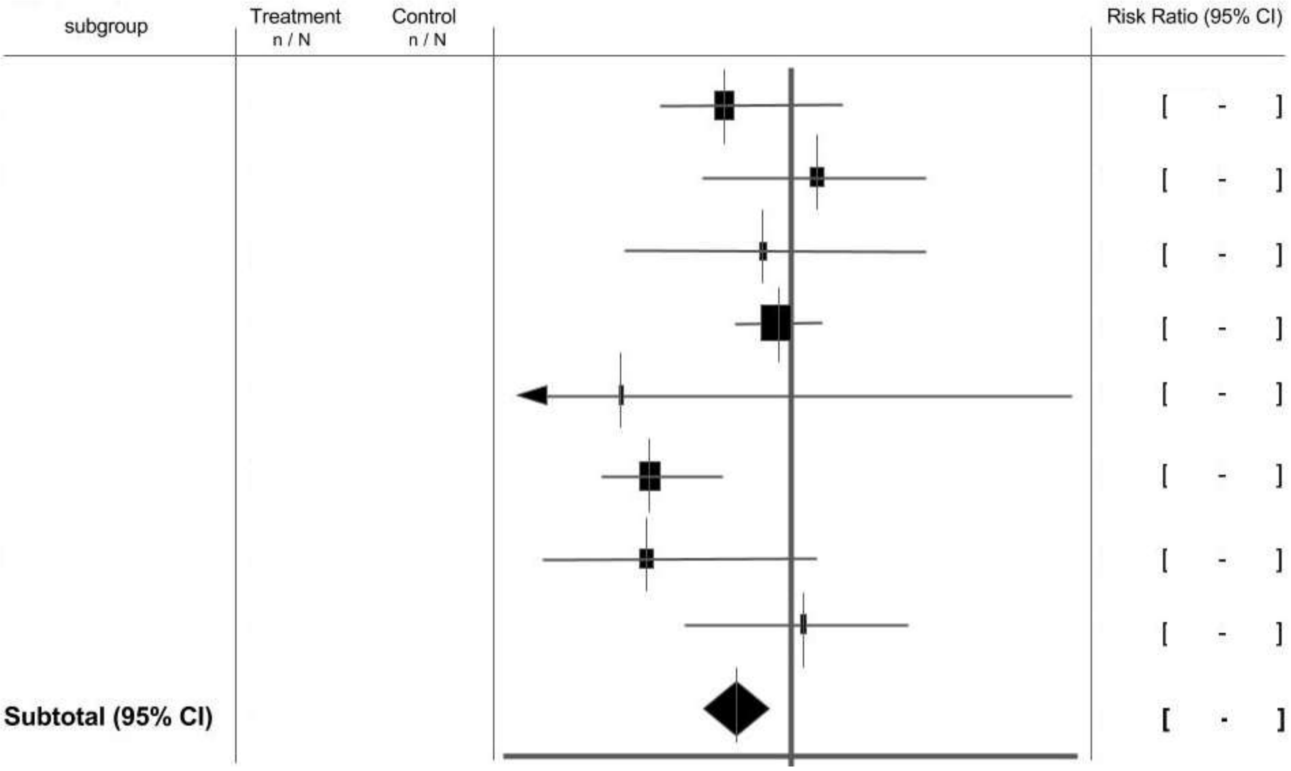

***To include:**

- **QRS Morphology (Dichotomised as Narrow QRS Duration/Non-Narrow QRS)**
- **PR Interval (Tertiles –Low vs Medium and Low vs High)**
- **Baseline Echocardiography (E/A Fusion)**
- **Selective vs Non-Selective His Bundle Pacing**
- **LV Lead vs His Lead**
- **Ejection Fraction Group (Dichotomised as ≤35[illegible]**
- **Device Type (Dichotomised as ICD vs non-ICD)**
- **BMI (Tertiles –Low vs Medium and Low vs High)**
- **Max RER Score (Tertiles - <0.95 vs 0.95-1.05, <0.95 vs >1.05)**
- **Height (Tertiles –Low vs Medium and Low vs High)**
- **Percentage His-pacing (dichotomised as ≤95% vs >95%)**
- **Adherence to optimal pacing based on metrics obtained at V4/V5 and AE data (binary)**

Safety Analysis Output

Table 7.1.1: Summary of Adverse Events by Treatment, Arm and Severity

		Pacing			No-Pacing		
		Arm 1	Arm 2	Total	Arm 1	Arm 2	Total
Severity	Mild						
	Moderate						
	Severe						
	Total						

Table 7.1.2: Summary of Adverse Events by Treatment, Arm and Relationship to Study

		Subjects with AEs*						
Treatment	Arm	No Relationship	Unlikely	Possible	Probable	Definitely	Not Yet Defined	Total
Pacing	1							
	2							
	Both							
No-pacing	1							
	2							
	Both							
All subjects								

		All AEs						
**Treatment**	**Arm**	**No Relationship**	**Unlikely**	**Possible**	**Probable**	**Definitely**	**Not Yet Defined**	**Total**
Pacing	1							
	2							
	Both							
No-pacing	1							
	2							
	Both							
All subjects								

*** For subjects with multiple AE's the highest-ranking AE will be used.**

Table 7.2.1: Summary of Adverse Device Effects by Treatment, Arm and Severity

		Pacing			No-Pacing		
		**Arm 1**	**Arm 2**	**Total**	**Arm 1**	**Arm 2**	**Total**
**Severity**	**Mild**						
	**Moderate**						
	**Severe**						
	**Total**						

Table 7.2.2: Summary of Adverse Device Effects by Treatment, Arm and Relationship to Study

		Subjects with AEs*						
**Treatment**	**Arm**	**No Relationship**	**Unlikely**	**Possible**	**Probable**	**Definitely**	**Not Yet Defined**	**Total**
Pacing	1							
	2							
	Both							
No-pacing	1							
	2							
	Both							
All subjects								
		**All AEs**						
**Treatment**	**Arm**	**No Relationship**	**Unlikely**	**Possible**	**Probable**	**Definitely**	**Not Yet Defined**	**Total**
Pacing	1							
	2							
	Both							
No-pacing	1							
	2							
	Both							
All subjects								

*** For subjects with AE's the highest-ranking AE will be used.**

Table 7.3.1: Summary of Serious Adverse Events by Treatment, Arm and Severity

		Pacing			No-Pacing		
		Arm 1	Arm 2	Total	Arm 1	Arm 2	Total
Severity	Mild						
	Moderate						
	Severe						
	Total						

Table 7.3.2: Summary of Serious Adverse Events by Treatment, Arm and Relationship to Study

		Subjects with SAEs*						
Treatment	Arm	No Relationship	Unlikely	Possible	Probable	Definitely	Not Yet Defined	Total
Pacing	1							
	2							
	Both							
No-pacing	1							
	2							
	Both							
All subjects								

		**All SAEs**						
**Treatment**	**Arm**	**No Relationship**	**Unlikely**	**Possible**	**Probable**	**Definitely**	**Not Yet Defined**	**Total**
Pacing	1							
	2							
	Both							
No-pacing	1							
	2							
	Both							
All subjects								

*** For subjects with multiple AE's the highest-ranking AE will be used.**

Table 7.4.1: Summary of Serious Adverse Device Effects by Treatment, Arm and Severity

		**Pacing**			**No-Pacing**		
		**Arm 1**	**Arm 2**	**Total**	**Arm 1**	**Arm 2**	**Total**
**Severity**	**Mild**						
	**Moderate**						
	**Severe**						
	**Total**						

Table 7.4.3: Summary of Serious Adverse Device Effects by Treatment, Arm and Relationship to Study

		Subjects with SAEs*						
**Treatment**	**Arm**	**No Relationship**	**Unlikely**	**Possible**	**Probable**	**Definitely**	**Not Yet Defined**	**Total**
Pacing	1							
	2							
	Both							
No-pacing	1							
	2							
	Both							
All subjects								
		**All SAEs**						
**Treatment**	**Arm**	**No Relationship**	**Unlikely**	**Possible**	**Probable**	**Definitely**	**Not Yet Defined**	**Total**
Pacing	1							
	2							
	Both							
No-pacing	1							
	2							
	Both							
All subjects								

*** For subjects with AE's the highest-ranking AE will be used.**

Table 7.5.1 –7.5.4: Mixed Effect Logistic Regression output to investigate proportional rate occurrence for Rates of Death/Hospitalisation/Revision of Device/Extraction or Removal of Device by treatment, period and sequence

Please refer to series of output in Table 4.2.

Table 7.6.1: Summary of Vital Signs Values

Variable*	Arm	Visit	Treatment	Samples Taken	n	Mean	SD	Median	Min.	Max.
	A	BL	-							
		M6	Control							
		M12	Treatment							
	B	BL	-							
		M6	Treatment							
		M12	Control							

***Variables to assess: Height, weight, pulse, BMI, Dia/Sys Blood Pressure Average**

Appendix A - Listings

Listing A: Listing of MVO2 values

Treatment	Subject	Site	Type	Start Date	End Date

Listing B: Listing of protocol deviations

Treatment	Subject	Site	Type	Start Date	End Date

Listing C: Listing of protocol violations

Treatment	Subject	Site	Type	Start Date	End Date

Listing D: Listing of Serious Adverse Events

Treatment: Pacing

Timepoint	Subj	AE Term	SAE Start Date	Ongoing	SAE End Date	Days frm Implant	Days frm Rand.	SAE Category	Expected-ness	Rel. to Study	Details

Treatment: No-Pacing

Timepoint	Subj	AE Term	SAE Start Date	Ongoing	SAE End Date	Days frm Implant	Days frm Rand.	SAE Category	Expected-ness	Rel. to Study	Details

Listing E: Listing of Serious Adverse Device Effects

Treatment: Pacing

Timepoint	Subj	AE Term	SAE Start Date	Ongoing	SAE End Date	Days frm Implant	Days frm Rand.	SAE Category	Expected-ness	Rel. to Device	Details

Treatment: No-Pacing

Timepoint	Subj	AE Term	SAE Start Date	Ongoing	SAE End Date	Days frm Implant	Days frm Rand.	SAE Category	Expected-ness	Rel. to Device	Details

REFERENCES

HOPE-HF Study Protocol v6.0 25Jan2018
